# Dynamics in the murine norovirus capsid revealed by high-resolution cryo-EM

**DOI:** 10.1371/journal.pbio.3000649

**Published:** 2020-03-31

**Authors:** Joseph S. Snowden, Daniel L. Hurdiss, Oluwapelumi O. Adeyemi, Neil A. Ranson, Morgan R. Herod, Nicola J. Stonehouse

**Affiliations:** Astbury Centre for Structural Molecular Biology, School of Molecular & Cellular Biology, Faculty of Biological Sciences, University of Leeds, Leeds, United Kingdom; Baylor College of Medicine, UNITED STATES

## Abstract

Icosahedral viral capsids must undergo conformational rearrangements to coordinate essential processes during the viral life cycle. Capturing such conformational flexibility has been technically challenging yet could be key for developing rational therapeutic agents to combat infections. Noroviruses are nonenveloped, icosahedral viruses of global importance to human health. They are a common cause of acute gastroenteritis, yet no vaccines or specific antiviral agents are available. Here, we use genetics and cryo-electron microscopy (cryo-EM) to study the high-resolution solution structures of murine norovirus as a model for human viruses. By comparing our 3 structures (at 2.9- to 3.1-Å resolution), we show that whilst there is little change to the shell domain of the capsid, the radiating protruding domains are flexible, adopting distinct states both independently and synchronously. In doing so, the capsids sample a range of conformational space, with implications for maintaining virion stability and infectivity.

## Abstract 2

High-resolution cryo-EM reveals that the norovirus capsid continuously samples conformational space, challenging the model of conformational changes in virus structure being orchestrated along linear, irreversible pathways of assembly and disassembly, with implications for infectivity and immune evasion.

## Introduction

High-resolution structural information has been key in improving our understanding of viral life cycles. However, viral capsids commonly undergo profound conformational changes during their infection cycles, as well as more subtle dynamics that can be challenging to capture. Understanding such conformational changes can hold the key for the design and development of antiviral agents and vaccines, which are needed for many viral diseases, including those caused by noroviruses.

Noroviruses are globally prevalent pathogens that cause approximately 200,000 deaths each year in low- and middle-income countries [1]. An effective vaccine or antiviral agent against norovirus would provide significant health and economic benefits, but none have been approved to date. Although several clinical trials of virus-like particle (VLP) vaccines have been undertaken, results have been disappointing [2,3]. This is partly because the available VLPs do not provide a sufficiently long-lived and appropriate immune response. It is therefore possible that the VLPs are not acting as an appropriate surrogate for the intact virion.

As members of the Caliciviridae family, norovirus virions consist of an approximately 7.5-kb positive-sense single-stranded RNA genome linked at its 5′ end to a viral protein (VPg). This genomic RNA is enclosed within a nonenveloped, *T* = 3 capsid approximately 40 nm in diameter [4,5]. Whilst there have been several high-resolution reconstructions of VLPs reported previously, structures of infectious noroviruses have been limited to a resolution of 8.0 Å until very recently [5–10]. The capsid is formed from 90 dimers of the major structural protein, VP1, in one of three quasiequivalent conformational states [11]: A-type VP1 proteins make up the icosahedral 5-fold axes and form AB dimers with B-type VP1 proteins, whereas C-type VP1 proteins form CC dimers at 2-fold axes. Each VP1 monomer comprises an N-terminal region, a shell (S) domain, and a protruding (P) domain (subdivided into proximal [P1] and distal [P2] subdomains). The S domains interact to completely enclose the contents of the capsid within an icosahedral shell. P domains extend outward from this shell and mediate interactions with receptor molecules and cofactors such as bile salts [12–14]. Located within the capsid interior are a limited number of molecules of the minor structural protein, VP2 [15]. Whilst VP2 is unresolved in all norovirus structures to date, structural studies with the related *Vesivirus*, feline calicivirus, showed that VP2 can form a portal-like assembly as a result of conformational changes caused by VP1 receptor engagement [16]. In doing so, it is believed to play an important role in viral genome release into the target cell.

Although the aforementioned structural studies have provided a wealth of information, there is still little knowledge on the dynamic nature of the viral capsid. Previously reported structures of noroviruses and related caliciviruses have revealed subtle differences within P domains, as well as dramatic conformational changes that alter the position of the P domain dimers relative to the S domains [6,7]. Currently, all structural information on human noroviruses comes from VLPs because of difficulties in culturing the infectious virus. Interestingly, infectious murine norovirus (MNV) apo structures demonstrate gross morphological differences compared to most of these human norovirus VLP structures [5,6,8,9]. Notably, an antibody-bound human norovirus VLP showed a conformation closer to that of MNV [17]. Recent structural studies of MNV in complex with various bile salts have indicated that bile salts can induce a contraction of the capsid and rotation of the P domains through an unknown mechanism [10]. Furthermore, an approximately 9.5-Å reconstruction of MNV in complex with its receptor, cluster of differentiation 300-like family member f (CD300lf), suggests that this ‘contracted’ state may be more favourable for receptor binding [10].

In this study, we present the high-resolution solution structure of infectious MNV. Unlike the previous reconstruction of apo wild-type MNV (wtMNV), our reconstruction is remarkably similar to the overall structure of most human norovirus VLPs and to structures of MNV complexed with the bile salts of glychochenodeoxycholic acid (GCDCA) and taurocholic acid (TCA) (despite the complete absence of bile salts in our structure) [10]. With an MNV reverse genetic system, we also study the stability and conformational flexibility of infectious norovirus, using in vitro evolution to generate a mutant virus with increased stability. Our analysis reveals that P domain dimers are independently mobile elements (i.e., they can move in a manner that is not coordinated with other P domain dimers) with the ability to sample a wide conformational space whilst maintaining infectivity. We hypothesise that this allows noroviruses to interact with a range of receptor molecules or cofactors and could improve antibody evasion. These are powerful selective advantages for viral growth and challenge the idea of viral capsids as static containers for their genomic RNA. This may pave the way for new ideas to generate better immune responses for vaccination or antiviral strategies.

## Results

### The cryo-electron microscopy reconstruction of wtMNV reveals dynamic P domains

**Fig 1:**
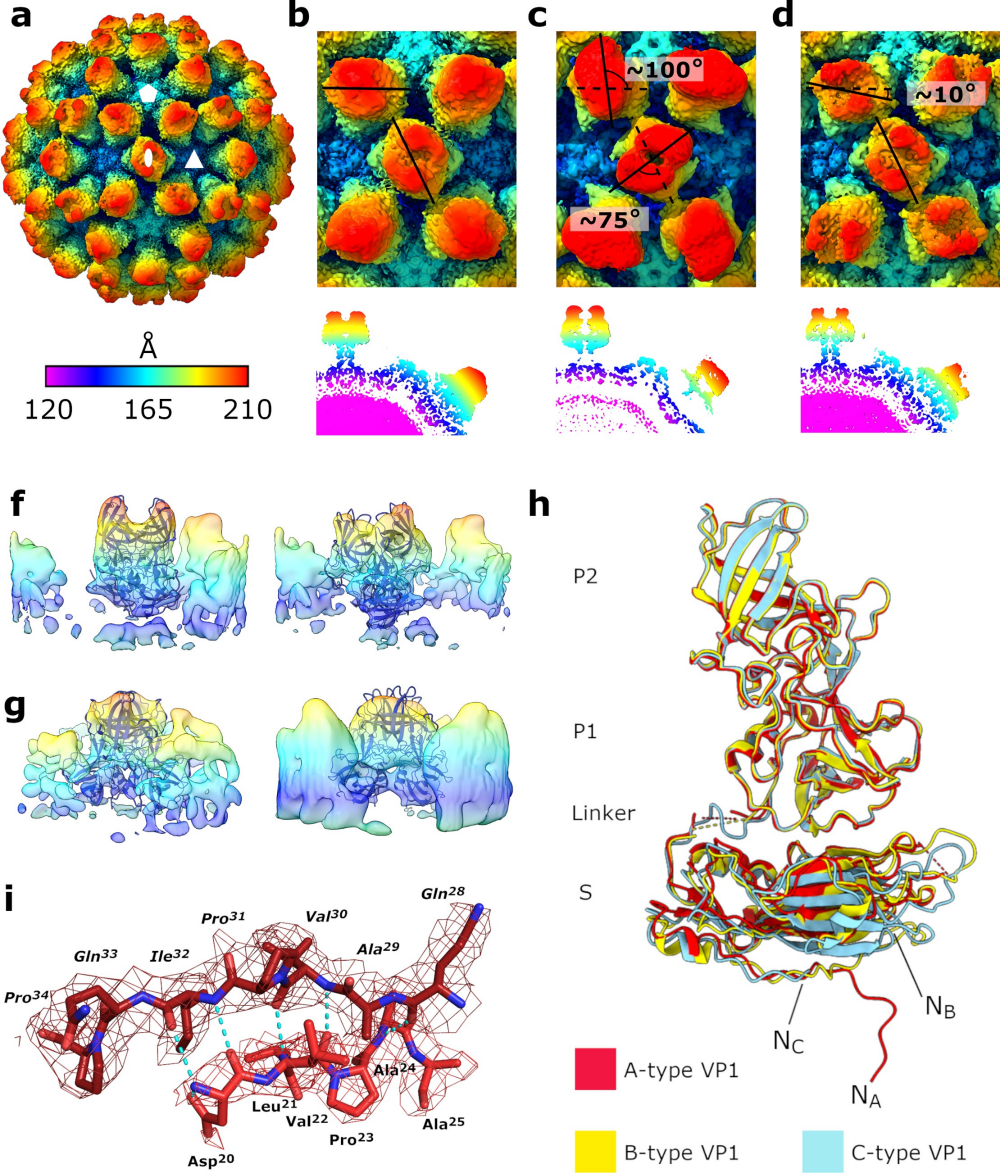
The 3.1-Å structure of wtMNV solved by cryo-EM. (a) Isosurface representation of the 3.1 Å wtMNV density map, shown at 1 σ and coloured according to the radial colouring scheme shown (Å). The 5-fold (hexagon), 3-fold (triangle), and 2-fold (oval) icosahedral axes are indicated. (b,c,d) Enlarged views centred on the icosahedral 2-fold axes of (b) our apo wtMNV reconstruction, (c) the recently published cryo-EM reconstruction of apo wtMNV from Sherman and colleagues [10], or (d) the reconstruction of wtMNV in complex with GCDCA reported by Sherman and colleagues [10]. All maps were low-pass filtered to 4.0 Å resolution and coloured according to the radial colouring scheme shown (Å). The angle of rotation for AB- or CC-type P domain dimers compared to the wtMNV reconstruction shown in (b) are given. Central sections through each reconstruction are also shown. (f,g) Side views of example class averages from focussed classification of (f) AB-type or (g) CC-type P domain dimers, with the atomic coordinates for wtMNV VP1 P domain dimers rigid-body fitted in each case. (h) Overlaid atomic coordinates for different quasiequivalent copies of MNV VP1, shown by the colouring scheme. The most N-terminal residue modelled for each quasiconformer is indicated by N_A_ (D20), N_B_ (S17), or N_C_ (V30). (i) Atomic coordinates for N-terminal regions of adjacent A-type VP1 molecules, with polar contacts shown with blue dashed lines. cryo-EM, cryo-electron microscopy; GCDCA, glychochenodeoxycholic acid; MNV, murine norovirus; P domain, protruding domain; S domain, shell domain; VP, viral protein; wt, wild type.

We used cryo-electron microscopy (cryo-EM) to determine the structure of wtMNV at 3.1-Å resolution (Fig 1A and S1 Fig). wtMNV was cultivated in RAW264.7 cells, then purified by ultracentrifugation through a sucrose cushion and 2 sucrose gradients before being applied to lacey carbon grids and vitrified. Cryo-EM data collection parameters are given in S1 Table.

As expected, the MNV capsid shows *T* = 3 icosahedral symmetry and is formed of 90 dimers of VP1. Comparison to a recently published cryo-EM reconstruction of apo MNV revealed striking differences in the position and orientation of P domains relative to the S domains [10] (S1 Movie). The P domains from AB-type dimers (surrounding the icosahedral 5-fold axes) are rotated approximately 100° clockwise relative to their position in the published structure [10], whilst the P domains from CC-type dimers (at icosahedral 2-fold axes) are rotated approximately 75° anticlockwise (Fig 1B and 1C). The P domains are also located much closer to the shell of S domains (separated by approximately 6 Å rather than approximately 16 Å). Interestingly, the positioning and orientation of the P domains in our map of apo MNV are closer (although not identical) to recent structures of bile-complexed MNV, despite the lack of bile salts [10]. In this case, CC-type dimers are in the same orientation, and AB-type dimers are rotated approximately 10° clockwise relative to our apo wtMNV (Fig 1D).

The lower resolution of the P domains (S2 Fig and S3 Fig) likely reflects the increased dynamics of these regions of the capsid. Reconstruction of a 3D density map from cryo-EM data involves the averaging of many images—if P domains are not rigidly held in the same position relative to the S domains, this averaging results in a blurring of density and loss of high-resolution information. The P2 subdomain, which is the furthest away from the highly rigid S domain, is particularly poorly resolved, limiting the regions of the P domain that can be modelled. This is especially problematic for AB dimers, suggesting that they are more mobile than CC dimers.

In an attempt to address the lower resolution of the P domains, we performed 3D classification on the wtMNV data set to investigate if this contained particles with P domains in a number of clearly defined orientations. Global 3D classification separated particles with better-resolved P domains (which were taken forward for the final icosahedral reconstruction) from those with extremely poorly resolved P domains (which were excluded from the final reconstruction). However, this approach did not reveal a class with a distinct conformation, which was different from that described above. This suggests that the lower resolution in this part of the map is a result of individual P domain dimer mobility and not the coordinated movement of P domains across entire capsids.

With this observation in mind, we performed focussed 3D classification on AB- and CC-type P domain dimers separately to investigate any difference (S4 Fig). This approach involves the assignment of 60 symmetrically redundant orientations to each particle and application of a mask to focus classification on a substructure within the virion—here, an AB- or CC-type P domain dimer. The assignment of symmetrically redundant orientations means that differences in the positioning of substructures that are purely a consequence of icosahedral symmetry (e.g., rotation of each AB dimer by 72° around the 5-fold axis) are accounted for and so would not be apparent in resulting classes. Rather, any variation between focussed classes is unrelated to the position of the substructure in the overall organisation of the capsid. A cylindrical mask was generated and centred on AB- and CC-type P domain dimers. This approach revealed a striking diversity in the orientation and location of AB dimers (Fig 1F and S4 Fig) but much less variation in CC dimer positioning (Fig 1G and S4 Fig). This rationalises the lower resolution of A- and B-type P domain density than that of C-type P domain density described above. Complete capsids reconstructed using orientation information from individual classes showed well-resolved density for the P domain dimer that was contained within the mask during focussed classification but lower quality density for P domain dimers outside of this mask (S4 Fig). Importantly, these data are consistent with P domain dimers being mobile elements that move independently of each other on the capsid surface. However, it should be noted that this approach did not lead to a significant improvement in the quality of P domain density, presumably because the number of particles contributing to the final reconstruction was split between multiple classes, reducing the data in any individual reconstruction. It should also be noted that one class (out of 10) for each of the AB- and CC-type dimer classifications was mirrored in the z-axis.

### An atomic model of wtMNV VP1

The quality of the data allowed us to build a hybrid atomic model for VP1 (S2 Table). To construct an initial atomic model for refinement into our EM density map, a homology model of an MNV VP1 S domain was generated using the Phyre2 server [18], based on the structure of the Norwalk virus capsid determined by X-ray crystallography (Protein Data Bank [PDB]: 1IHM) [9]. This homology model was rigid-body fitted into our density map, and copies were fitted into each quasiequivalent position (A, B, and C) within the asymmetric unit of the icosahedral capsid. The fit against the experimental cryo-EM density was visually inspected, and any obvious deviations were corrected before the atomic coordinates were refined to improve fit and the geometry of the model. The resolution in the S domain was sufficient to allow confident building of most residues up to the flexible linker region. As expected, the flexible linker connecting the S and P domains was not resolved for A- or B-type VP1. However, the flexible linker for C-type VP1 was resolved, suggesting it is less mobile than the A- or B-type conformer, which correlates with the improved resolution of C-type P domains. To model the P domains, a crystal structure of an MNV VP1 P domain (PDB: 6C6Q) [13] was fitted into each quasiequivalent position, then refined against the cryo-EM density in combination with the S domain model, with secondary structure restraints imposed.

Comparison with a recent structure of MNV + bile salt (GCDCA) (PDB: 6P4J [10]) revealed that the P domains occupied broadly similar positions relative to the S domains (though AB-type dimers are rotated by approximately 10°). However, there is a different relative B-factor distribution than for the apo VP1 reported here, particularly in the P1 subdomains (S5 Fig). Furthermore, the flexible linkers for all quasiconformers of VP1 in the structure of MNV + GCDCA (PDB: 6P4J) are resolved [10].

We observed subtle but important differences between VP1 molecules occupying A-, B-, and C-type quasiequivalent positions within the asymmetric unit (Fig 1H). Quasiconformers were aligned based on a subset of close-matching atom pairs, and then root mean-square deviation (RMSD) values between the CA atoms of each VP1 monomer were calculated. RMSD values between the subset of residue pairs used for alignment of the fitted atomic coordinates were small (0.87 Å [A–B], 0.79 Å [A–C], 0.75 Å [B–C]) but increased when all residue pairs present in each of the quasiconformers being compared were included after alignment (2.77 Å [A–B], 2.46 Å [A–C], 2.35 Å [B–C]). This reflects a high degree of overall similarity between VP1 molecules but with certain regions showing substantial variability. In particular, there are striking differences between the N-terminal regions of the 3 quasiconformers. Most of the C-type VP1 N-terminal region is disordered (residues 1–29), whereas A- and B-type VP1 N-terminal regions are better resolved (missing residues 1–19 and 1–16, respectively) and have distinct conformations. The B-type N-terminal region remains close to the underside of the S domain and turns to run towards the icosahedral 3-fold axis. Comparatively, the A-type N-terminal regions protrude deeper into the capsid to interact with adjacent A-type N-terminal regions around the icosahedral 5-fold axis (Fig 1I). In summary, these data show both the defined nature of VP1 quasiconformers and the dynamic nature of the P domains.

### Thermal inactivation of wtMNV results in intact, noninfectious virus particles

Given the dynamic nature of the virion, we next aimed to capture in vitro-defined conformations of the MNV virion that reflect the conformational changes a norovirus capsid undergoes during the viral life cycle. For enteroviruses, thermal stressing is an established approach to inducing alternative capsid conformations that are informative of those that occur during cell entry [19,20]. We therefore applied this approach to MNV in order to identify structural changes that occur to the norovirus capsid after thermal stress. Our hypothesis was that more mobile elements of the viral capsid would be the first to change conformation upon heating.

**Fig 2:**
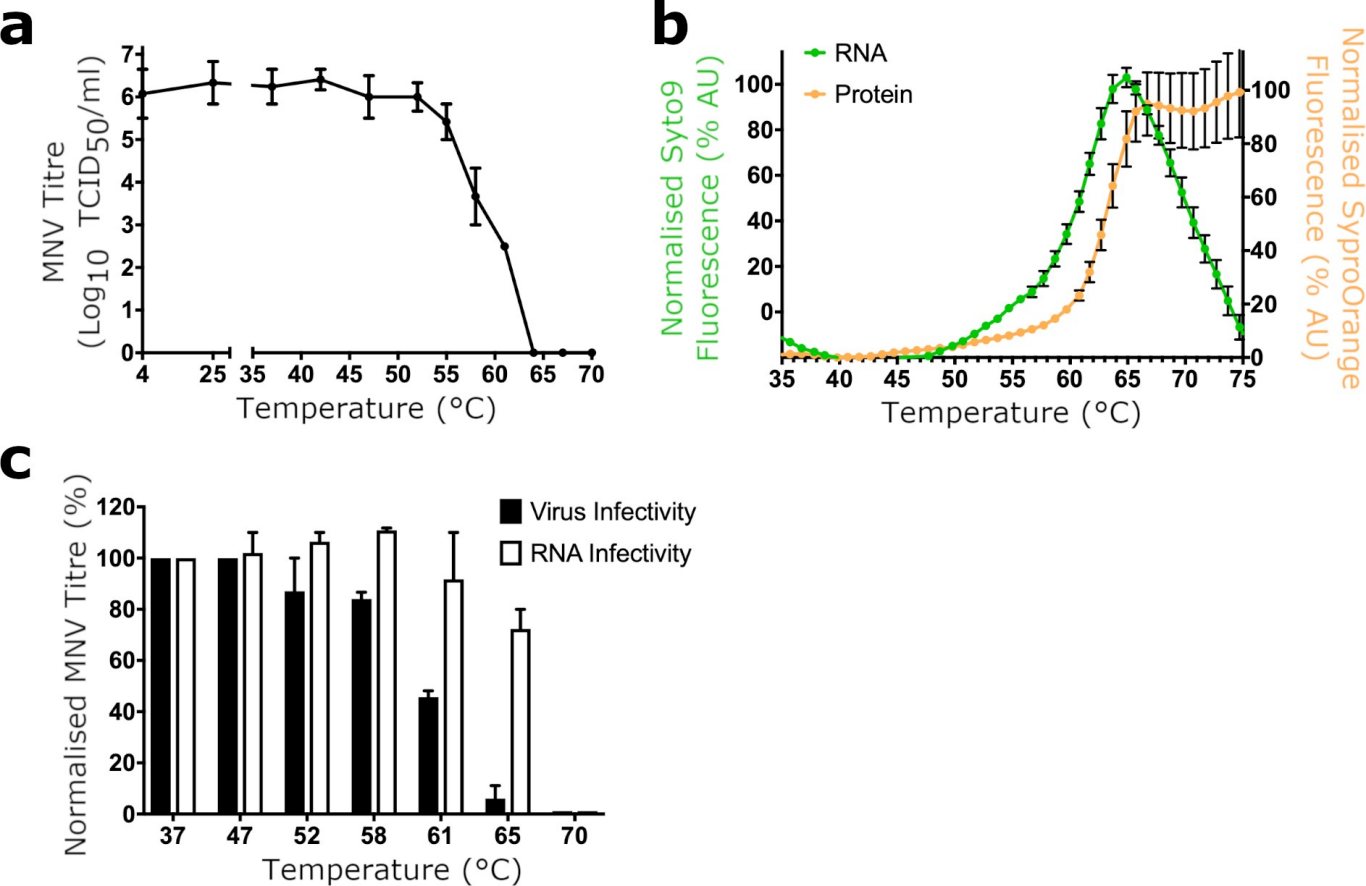
Thermal inactivation of wtMNV. (a) Samples of wtMNV were incubated at a range of temperatures up to 70°C on a 30-second constant temperature ramp before being immediately cooled on ice. Titres were determined by TCID_50_ assay on RAW264.7 cells (*n* = 2 ± SEM). (b) wtMNV was purified by sucrose density gradient, dialysed into PBS, and used for PaSTRy thermal stability assays using the nucleic acid dye SYTO-9 (green) and the protein dye SYPRO-Orange (orange) on a 30-second constant temperature ramp (*n* = 3 ± SEM). (c) Samples of MNV were heated to the indicated temperatures, treated with RNase A, and then titrated by TCID_50_ assay on RAW264.7 cells (*n* = 2 ± SEM) or used to extract total RNA. The extracted RNA was transfected into BHK cells (which only permit a single round of replication), and resultant virus was harvested and titrated by TCID_50_ assay on RAW264.7 cells (*n* = 2 ± SEM). Numerical data for Fig 2 are provided in S1 Data. BHK, baby hamster kidney; MNV, murine norovirus; PaSTRy, Particle Stability Thermal Release; PBS, phosphate-buffered saline; TCID_50_, median tissue culture infectious dose; wt, wild type.

This first required us to characterise the thermal stability of MNV virions. We therefore performed median tissue culture infectious dose (TCID_50_) assays with MNV after heating on a 30-second constant temperature ramp to identify a point at which the virus had lost >99.9% titre (Fig 2A). We also investigated capsid stability by performing Particle Stability Thermal Release (PaSTRy) assays, which employ 2 fluorescent dyes, SYTO-9 (which binds to nucleic acids) and SYPRO-Orange (which binds to hydrophobic regions of proteins), to assess the stability of viral capsids independently of viral infectivity [21] (Fig 2B). Whilst there was a 99.99% reduction in infectivity at 61°C, PaSTRy assay data suggested that capsids remained essentially intact up to approximately 64°C because minimal SYTO-9 fluorescence suggests that the viral RNA was not exposed to bulk solvent below this temperature. Confirming this, MNV heated to 61°C (which we termed heat-inactivated MNV, or hiMNV) was incubated with RNase, and no digestion of the RNA genome was observed (Fig 2C). Thus, we had identified a temperature at which the capsids had become irrevocably noninfectious but were not disassociated into their component parts.

### The cryo-EM reconstruction of hiMNV reveals an increase in P domain mobility

To understand the structural changes that occurred during thermal stress, we determined the structure of hiMNV by cryo-EM at 2.9-Å resolution. This resolution is higher than for wtMNV (2.9 Å versus 3.1 Å; S1 Fig, S2 Fig and S3 Fig), although considerably more data were available for the hiMNV reconstruction (i.e., more particles were used to generate the final reconstruction). There were no gross morphological differences in the positioning of the P domains with respect to the S domains compared to the wtMNV map.

**Fig 3:**
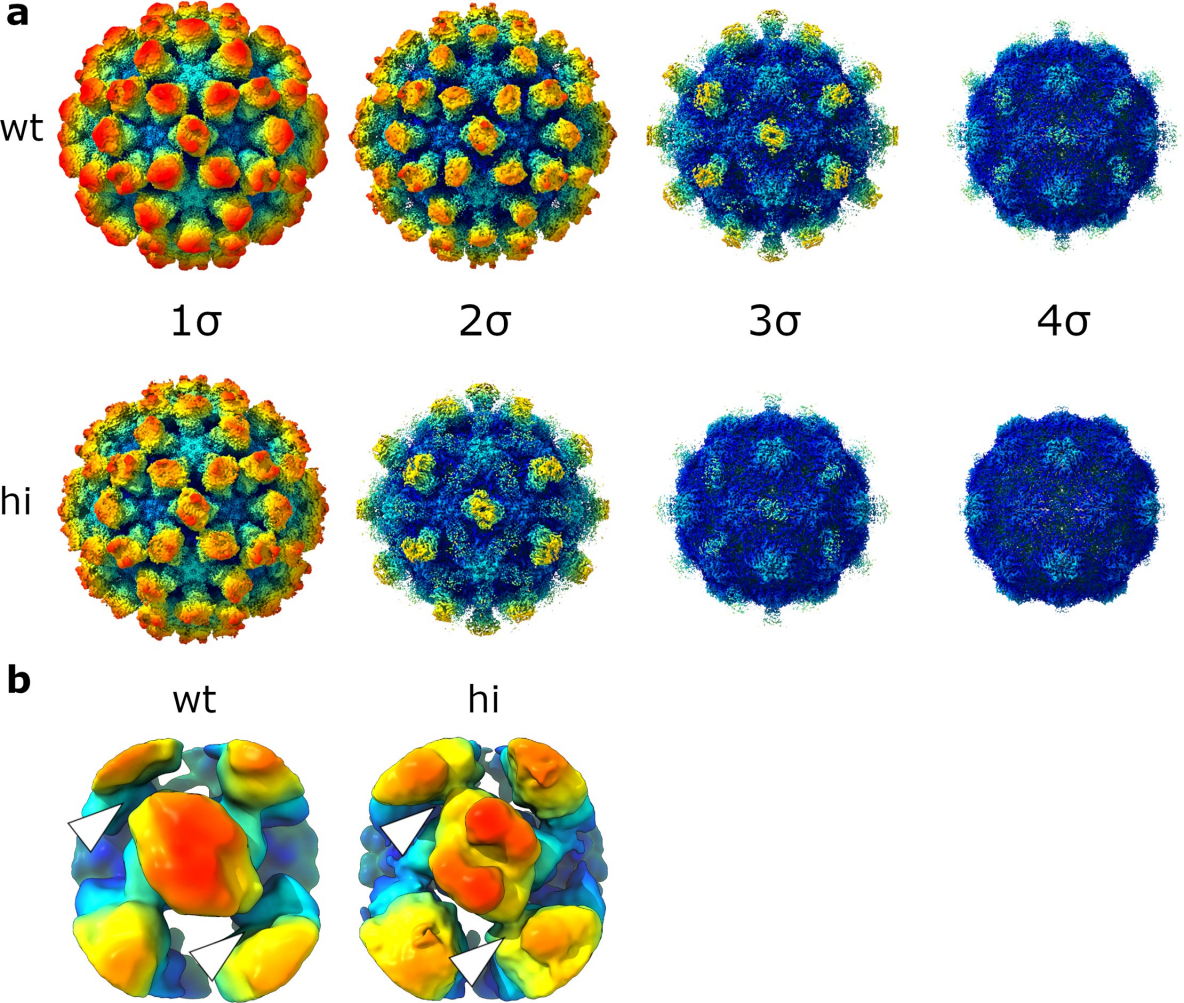
hiMNV has weaker P domain density than wtMNV. (a) Isosurface representations of the 3.1-Å wtMNV reconstruction (wt) and the 2.9-Å hiMNV reconstruction (hi), contoured to different thresholds (1 σ–4 σ). (b) Example focussed classes from focussed classification of wtMNV and hiMNV CC-type P domain dimers, shown at 2 σ. The sites of contact between C-type and A-type P domains, used to determine ‘noncontacting’ status, are indicated by white arrowheads. All focussed classes are shown in S4 Fig (wtMNV) and S6 Fig (hiMNV). hiMNV, heat-inactivated MNV; MNV, murine norovirus; P domain, protruding domain; wt, wild type.

Furthermore, whilst the strength of the S domain density was similar between the 2 maps, P domain density was weaker for hiMNV than for wtMNV (Fig 3A). This effect could result from either the P domain itself becoming more flexible (or a subpopulation of P domains being denatured by the heat treatment) or the P–S linkage becoming more mobile and thus the P domains occupying a larger range of positions relative to the shell.

To further investigate the effect of heat treatment on the P domains, we performed focussed classification. We undertook this on the hiMNV CC-type P domain dimers because these were better resolved than AB-type dimers. Interestingly, all of the classes showed the central CC-type dimer making contacts with adjacent AB-type dimers. This is in comparison to wtMNV, in which approximately 8.0% of CC-type dimers populated a class with no interactions between CC- and AB-type dimers, which we termed a ‘noncontacting’ class (Fig 3B, S4 Fig and S6 Fig). This suggested that heat treatment had changed the conformational landscape explored by the P domains (i.e., the range of conformations accessible to the P domains), which may be related to the loss of infectivity. Whilst hiMNV focussed classification gave several ‘junk’ classes with poor-quality P domain density (suggesting that a subpopulation of P dimers could have been denatured by heating), only a minority of P domain dimers were assigned to these classes.

### Selection of a heat-stable mutant MNV

**Fig 4:**
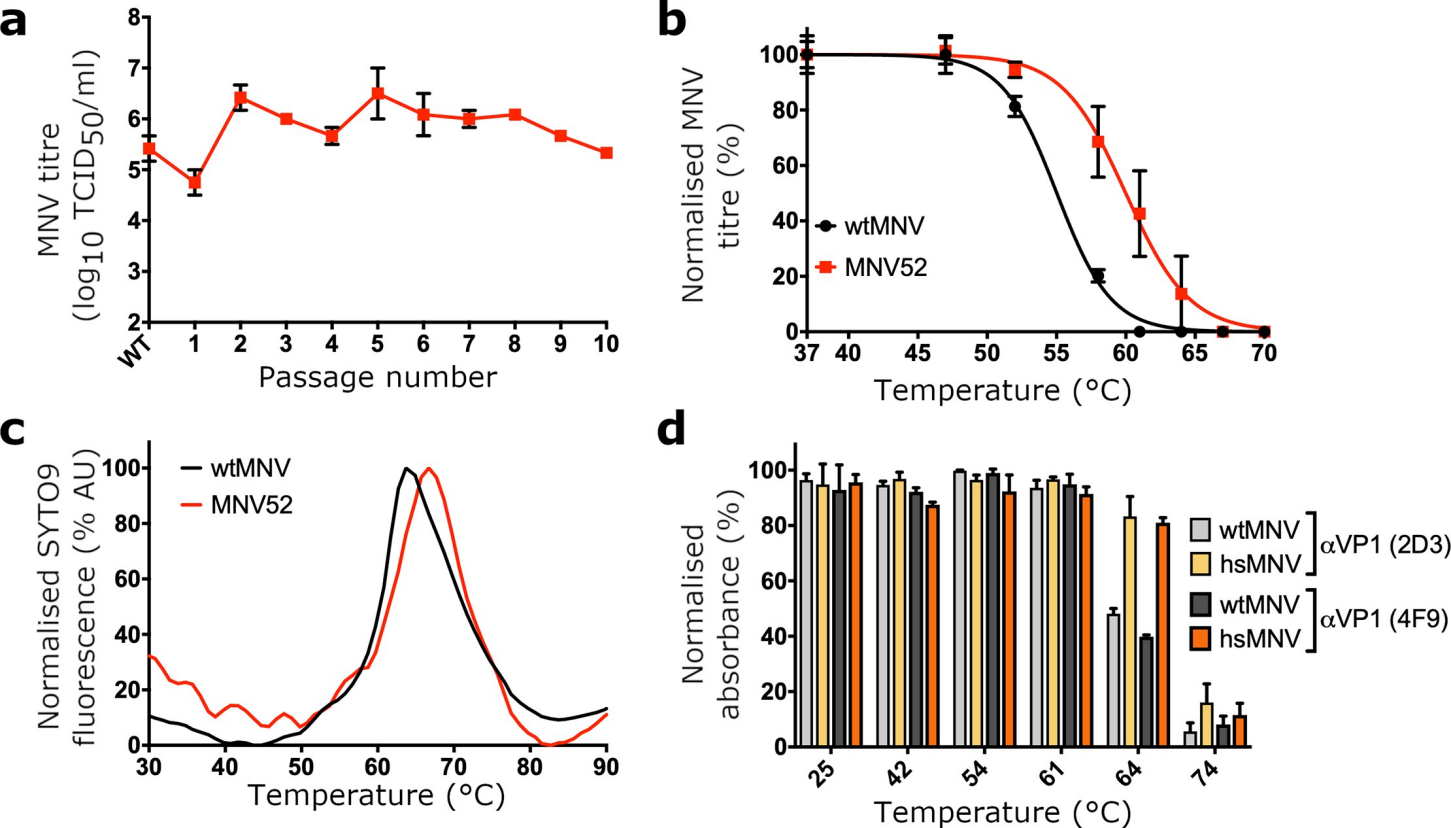
Selection and biochemical characterisation of hsMNV. (a) Samples of MNV were heated at 52°C for 30 min before cooling to 4°C. The surviving pool of viruses was subsequently passaged for 48 hours at 37°C on RAW264.7 cells. Prior to selection (‘wt’) and at each passage, the virus titre was determined by TCID_50_ on RAW264.7 cells (*n* = 2 ± SEM). Consecutive cycles of selection were performed. (b) The pool of virus heated at 52°C between passages (termed MNV52) and wtMNV were heated to a range of temperatures between 37°C and 70°C and virus titre determined by TCID_50_ assay on RAW264.7 cells (*n* = 4 ± SEM). (c) Virus samples were purified by sucrose density gradient, dialysed into PBS, and used for PaSTRy thermal stability assays using the nucleic acid dye SYTO-9. (d) The antigenicity of wtMNV or hsMNV was determined by ELISA with anti-VP1 antibodies, 2D3 and 4F9, after incubation at the indicated temperature (*n* = 2 ± SD). Numerical data for Fig 4 are provided in S2 Data. hsMNV, heat-stable MNV; MNV, murine norovirus; PaSTRy, Particle Stability Thermal Release; PBS, phosphate-buffered saline; TCID_50_, median tissue culture infectious dose; VP, viral protein; wt, wild type.

Together, our data thus far suggest that P domains are highly mobile elements; however, our observations with hiMNV suggest that greater mobility is inversely correlated to infectivity. Therefore, it would follow that mutant viruses with improved thermostability would have mutations specifically affecting VP1 P domain conformation or mobility. To acquire a genetic insight into the structural determinants of P domain mobility, we generated a thermally stabilised mutant MNV by in vitro evolution. A thermally stabilised mutant MNV was isolated by repeated cycles of selection at 52°C (Fig 4A). This pool of virus, termed MNV52, showed improved thermal stability compared to wtMNV, as anticipated (Fig 4B). A PaSTRy assay revealed that viral RNA became exposed from the capsid at temperatures above that recorded for wt (Fig 4C), consistent with the data in Fig 2B.

**Fig 5:**
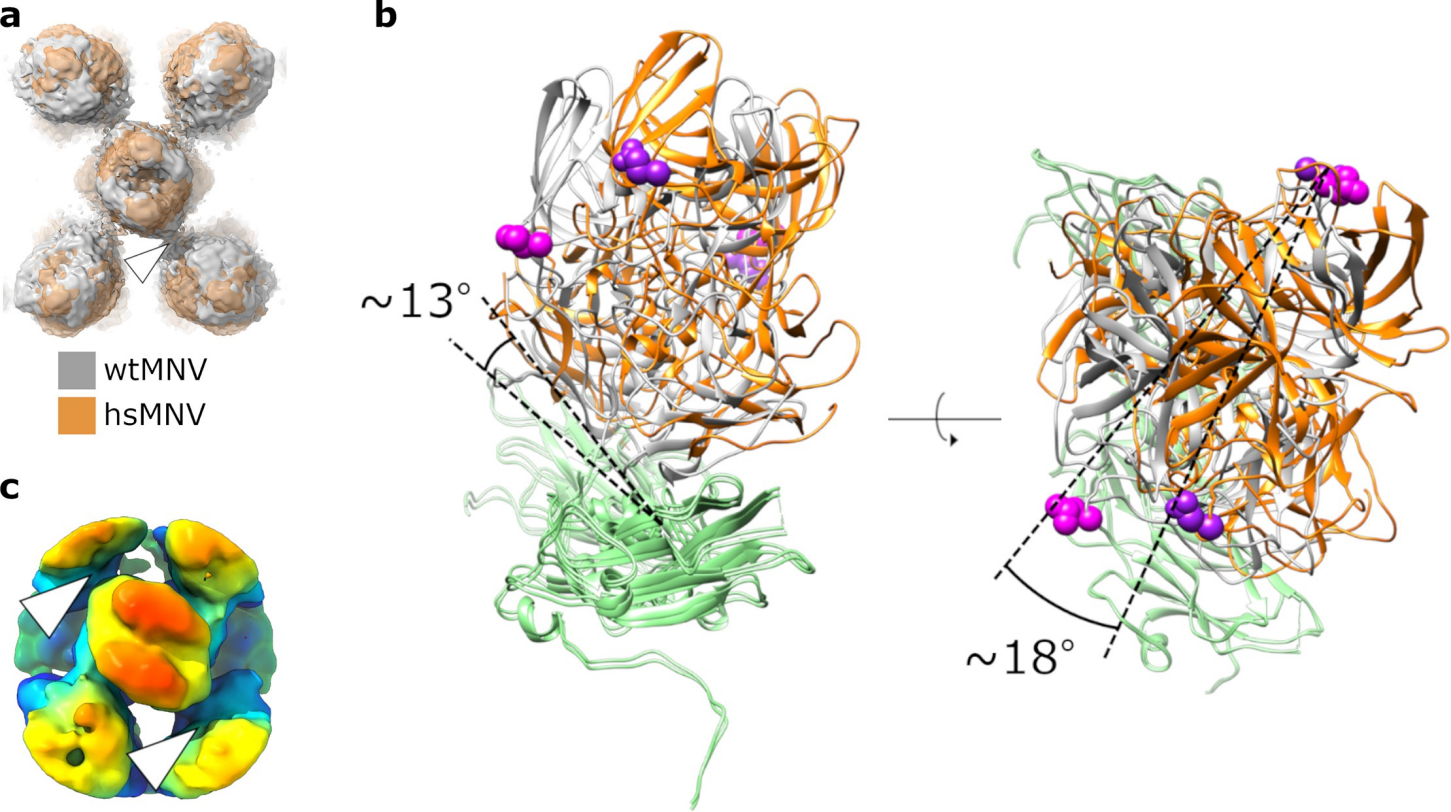
hsMNV has ‘twisted’ P domain dimers relative to wtMNV. (a) Overlaid isosurface representations of wtMNV (grey) and hsMNV (orange) centred on the icosahedral 2-fold axis, shown at 1 σ after low-pass filtering both maps to the same resolution (4.0 Å). The back plane is clipped to remove the S domains. (b) Atomic coordinates for AB-type VP1 fitted into wtMNV (grey) or hsMNV (orange) density maps, centred on the AB dimer–CC dimer interface highlighted by the white arrowhead in (a). S domains are shown in green. The mutated residue is shown as magenta (wtMNV, L412) or dark purple (hsMNV, L412Q) spheres. Angles of rotation in 2 axes are indicated. (c) A ‘noncontacting’ class resulting from focussed classification of hsMNV CC-type P domains, shown at 2 σ. hsMNV, heat-stable MNV; MNV, murine norovirus; P domain, protruding domain; S domain, shell domain; VP, viral protein; wt, wild type.

To identify mutation(s) present in the thermostable MNV population, the structural protein-encoding region of the MNV genome (ORF2 and ORF3) was amplified by reverse transcriptase (RT)-PCR and sequenced at the consensus level. No mutation was seen in ORF3 (encoding VP2), but a single mutation was found in ORF2 that leads to a single amino acid substitution in VP1, L412Q. Consistent with our hypothesis, this mutation was located in the VP1 P domain, on the hinge loop connecting the P1 and P2 subdomains (for reference, see Fig 5B). To further characterise the effect of the L412Q substitution, we reconstituted the mutation in an infectious clone of MNV and used it to recover ‘heat-stable’ (hs)MNV particles. Like MNV52, hsMNV remained infectious after incubation at temperatures that rendered wtMNV noninfectious (S7 Fig). Given that hsMNV had an amino acid substitution in the P domain of VP1, we also looked for changes in antigenicity by ELISA that may be indicative of a conformational change. We investigated binding to 2 neutralising anti-VP1 antibodies, 2D3 and 4F9 [22]. This showed that the mutant retained 2 major epitopes, including at 64°C (a temperature that wtMNV could not tolerate) (Fig 4D).

### The cryo-EM reconstruction of hsMNV shows ‘twisted’ AB-type P domains

To gain a structural insight into the mechanism of stabilisation of hsMNV, we determined the structure of hsMNV to a resolution of 3.1 Å (S1 Fig, S2 Fig and S3 Fig). Interestingly, whilst CC-type P domain dimers appear virtually identical to wtMNV, AB-type P domain dimers showed a subtle difference in their orientation (Fig 5A and S2 Movie). To explore this change, we performed rigid-body fitting of the atomic coordinates for wtMNV VP1 into the hsMNV map. This was followed by refinement against the hsMNV map with secondary structure restraints enabled. In the icosahedrally averaged wtMNV map, an interface is formed between A-type and C-type P domains, but for hsMNV, this interface has been disrupted (S7 Fig). Whilst the C-type P domain did not show any significant movement, the AB-type P domain dimer has tilted upwards by approximately 13°, angling away from the S domains, and rotated in an anticlockwise direction by approximately 18° (Fig 5B and S3 Movie). As such, the mutated residue now points away from the interface. In agreement with this, PDB in Europe Protein Interfaces, Surfaces and Assemblies (PDBePISA) [23] analysis of VP1 fitted into the wtMNV map suggests that C-type VP1 L412 is a buried residue and contributes to an interface with A-type VP1. When fitted into the hsMNV map, no interface is detected between A-type and C-type VP1.

Interestingly, focussed classification of CC-type P domain dimers for hsMNV revealed approximately 13.3% of P domain dimers populating ‘noncontacting’ classes (i.e., in which the CC-type P dimer makes no contact with adjacent AB-type dimers at the site of the L412Q mutation) (Fig 5C and S7 Fig). This is in contrast with wtMNV (approximately 8.0%) and hiMNV (for which there are no ‘noncontacting’ classes) (S4 Fig and S6 Fig).

It is possible that the heat-stable phenotype may be a consequence of the mutation stabilising the P domain fold, making it resistant to denaturation upon heating. However, when taken together, our data suggest that the conformational landscape explored by the P domain dimers of the mutant virion has changed, allowing it to retain a balance between P domain mobility and flexibility at elevated temperatures.

## Discussion

The structural data presented above describe the dynamic nature of an MNV virion by comparing 3 high-resolution structures of a single viral species. We showed that the infectious virion is a macromolecular complex with highly flexible P domains that are capable of sampling a range of conformational space whilst maintaining functionality. However, it appears from the increased P domain flexibility of heat-inactivated virions that too much mobility is detrimental to infectivity. Therefore, it seems that the virus has evolved to establish a balance between too much and too little P domain mobility in order to generate an optimal conformational landscape for the P domains to explore.

MNV is a genogroup V norovirus and so is related genetically and structurally to human noroviruses (genogroups I, II, and IV), and the well-established cell culture and reverse genetic systems allowed the functional significance of our structural data to be probed [24,25]. Using this system, we report the high-resolution solution structure of an infectious norovirus. Our cryo-EM reconstruction of wtMNV is strikingly different to the published apo structures of wtMNV [6,10]. Our structures show large changes in the positioning and orientation of P domain dimers relative to the S domains, as well as an approximately 10-Å difference in the distance between P and S domains [6,10]. These gross changes were consistent across all 3 of our independent reconstructions (discussed below). Importantly, the orientation and positioning of P domains in our reconstructions are consistent with all but two human norovirus VLPs [5,9].

Currently, the reasons for the differences between our wtMNV reconstruction and the previously published apo MNV structures are unclear. Given the flexible nature of the P domains demonstrated by our work, it is plausible that the 2 structures represent different functionally relevant MNV capsid conformations. These could have been induced experimentally (e.g., during virus purification, buffer conditions, or handling), and we cannot exclude subtle differences in the VP1 primary sequences (e.g., arising from mutation during passage), despite both starting with a CW1 strain of MNV-1 [7]. Given the changing ionic composition of the environment that the virus is exposed to during endocytosis [26], these subtle experimental details may be of biological importance; e.g., it has been shown that Ca^2+^ and Mg^2+^ (included in the buffer used here but not in the buffers used by Katpally and colleagues or Sherman and colleagues) can enhance P domain–receptor interactions, with one suggested mechanism being stabilisation of the P domain [6,10,13]. In contrast, Zn^2+^ ions, which have been implicated in norovirus VLP stability [8], are absent in the buffer used in this study. We do not see any clear metal ion density in our reconstructions, although this analysis is complicated by the poor resolution at the extremities of the P domains.

Intriguingly, our apo structure is similar to the bile-salt–complexed MNV structures reported recently, despite the absence of bile salts in our sample [10]. Notably, both GCDCA and TCA induced the collapse and rotation of P domains in line with our structure, but TCA was previously reported as failing to bind to MNV P domains [13]. Sherman and colleagues suggest that the bile salts may be acting as kosmotropes, stabilising water–water interactions, which would in turn stabilise intramolecular interactions in VP1 [10]. If this is the case, perhaps another component of the buffers used here may have also acted as a kosmotrope.

The independent mobility of individual P domain dimers on the viral capsid surface that we have observed is striking. It has been hypothesised that P domain conformation may differ within an individual norovirus species [27]. Here, we complement recent evidence from genogroup II norovirus VLPs and bile-salt–conjugated MNV [8,10] by providing direct structural evidence for such alternative morphologies within a single species as well as within individual norovirus particles. Using focussed 3D classification, we identified a remarkable diversity in P domain dimer positioning that was not coordinated over the capsid surface and was not equal between different quasiconformers, with CC-type dimers being less mobile than AB-type dimers. This contrasts with published data for 2 other caliciviruses, rabbit haemorrhagic disease virus and Tulane virus, in which CC-type dimers were shown to be more mobile [6,28]. Interestingly, it has previously been shown that a monoclonal antibody is able to bind to a site that is occluded in a human norovirus VLP structure, suggesting that alternate capsid morphologies are also likely to exist in human noroviruses [17].

We found a high degree of variability between the N-terminal regions of quasiequivalent VP1 subunits, corroborated by the structures of wtMNV in the presence of bile salts, which were published during the preparation of this manuscript [10]. These differences could provide a mechanism to position other components of the virion such as VP2, VPg, or the genome. However, in line with other norovirus structures, we cannot locate VP2 in any of the structures reported here. Based on recent work with feline calicivirus, it has been proposed that VP2 may bind the capsid interior at a single icosahedral 3-fold axis [29]. Whilst it has been shown through mutational analysis that VP1 N-termini are not required for coprecipitation with VP2 [15], the ability of VP1 N-termini to organise differently may provide an interface for ‘recognition’ of the 3-fold axis (formed by B- and C-type VP1 subunits) either directly or indirectly, e.g., VPg, which in turn guides VP2.

To support the hypothesis that P domains are mobile elements that can adopt multiple conformations, we induced conformational changes by thermal stress, generating intact noninfectious capsids (hiMNV). Cryo-EM studies on hiMNV revealed no gross morphological differences but weaker P domain density compared to wtMNV. In theory, weaker density may arise from ‘partial occupancy’ (i.e., inclusion of particles that have lost or denatured P domains in the final reconstruction) or be a result of increased P domain flexibility/mobility. In our data of hiMNV, several CC-type P-dimer focussed classes had poor-quality density, indicating that a subpopulation of dimers had denatured. However, we find no evidence to suggest that particles had lost P domains despite observing some small particles in raw micrographs from this data set (S1 Fig). Therefore, the weaker P domain density is more likely to be a result of increased P domain flexibility/mobility. This is supported by the assignment of most CC-type P dimers to focussed classes with good-quality density and that there were no P-domain–lacking capsids (S6 Fig). We speculate that increased P domain flexibility is detrimental to infectivity by changing the range of conformations sampled such that a conformation required for infection (e.g., for virus–receptor interactions) is inaccessible. In support of this idea, hiMNV had no ‘noncontacting’ P domain classes, suggesting heating had indeed changed the range of conformations that P domain dimers were able to adopt. However, we cannot rule out the possibility that other subtle changes within the unresolved regions of our map or the unresolved virion components (e.g., VP2 or VPg) contribute to virus inactivation.

To support the insights provided by cryo-EM on hiMNV, we passaged MNV with a thermal selection pressure in order to acquire resistance to heat inactivation. The thermally stable virus contained a single point mutation (L412Q) at the consensus level in the gene encoding VP1 (ORF2). Our cryo-EM reconstruction of hsMNV showed that AB-type P domain dimers were ‘twisted’ relative to wtMNV and tilted away from the S domain surface, disrupting an interface between AB- and CC-type dimers. It may not be immediately obvious why the mutant morphology is more stable—one would expect disruption of an interface to decrease stability. Indeed, thermostabilised foot-and-mouth disease virus (FMDV) capsids were generated by stabilising the interface between adjacent pentamers [30], and many stabilising mutations identified in poliovirus likely act by stabilising subunit interfaces [31]. Accordingly, it is plausible that the mechanism of stabilisation of the L412Q mutation is through stabilisation of the fold of the P domain itself against denaturation. However, we suspect that the ‘twisted’ morphology of hsMNV allows P domain dimers to enter a protective conformation upon heating, which may only become apparent at high temperatures. In support of this, a greater proportion of CC-type P domain dimers were assigned to ‘noncontacting’ classes than for wtMNV, suggesting that the conformational landscape explored by P domains has changed in the opposite direction to that induced by heat treatment (hiMNV).

In addition to providing insights about fundamental virus biology, hsMNV also offers a platform for the development of a thermostabilised vaccine against noroviruses. Most norovirus vaccine candidates under development are VLPs [32]. VLPs lack a viral genome, which can provide stabilising interactions with the capsid [33]. Thus, VLPs can be inherently unstable antigenically, which may be a problem during vaccine distribution. Norovirus vaccines are most urgently needed in countries with warmer climates and require a cold chain, which is particularly challenging to maintain in hard-to-reach regions, so stabilised vaccines are a particularly attractive prospect. By incorporating mutations (e.g., L412Q) into VLP production, it may be possible to generate stable VLPs. It is essential for this strategy that mutant VLPs generate an appropriate neutralising antibody response. In this regard, MNV carrying the L412Q mutation (hsMNV) retained its major antigenic determinants when tested against 2 neutralising antibodies. Furthermore, VP1 sequences from human noroviruses (genogroups I, II, and IV) show conservation of a hydrophobic residue in this position (S8 Fig). Thus, it is plausible that similar mutations may stabilise human norovirus VLPs as immunogens.

## Materials and methods

### Cell lines and antibodies

RAW264.7 cells (kindly gifted by Ian Clarke, University of Southampton) and BHK-21 cells (obtained from the ATCC; Manassas, VA, USA) were maintained in high-glucose Dulbecco’s Modified Eagle’s Medium (DMEM) supplemented with 10% (v/v) foetal bovine serum, 20 mM HEPES buffer, and 50 U/ml penicillin and streptomycin. Cells were incubated at 37°C, 5% CO_2_. Neutralising antibodies against MNV VP1, 2D3 and 4F9 [22], were kindly gifted by Christiane Wobus (University of Michigan).

### MNV propagation

To generate virus for use in this study, MNV-1 strain CW1P3 [34] (referred to as MNV) was recovered from an infectious clone, and propagated in RAW264.7 cells as described previously [24]. Briefly, RAW264.7 cells were seeded in T175 flasks and allowed to reach 80% confluency. They were then infected with crude stocks of MNV in fresh media and incubated for 48–72 hours, then harvested when confluent cytopathic effect was visible. The infectious media was put through 3 freeze–thaw cycles to release virus. To concentrate virus for propagation at high multiplicity of infection (MOI), the infectious lysate was first clarified (3,300 × *g*, 10 min, 4°C), then taken for ultracentrifugation at 366,000 × *g* (60 min, 4°C), and the pellet was resuspended in phosphate-buffered saline (PBS).

To minimise the chance of reversion, concentrated crude stocks of hsMNV were heated to 52°C for 30 min between each passage. Both wtMNV and hsMNV were validated by sequencing prior to structural analysis.

### MNV purification

To purify MNV, we followed modified versions of the protocols described by Hwang and colleagues [35]. Infectious media were collected and freeze–thawed 3 times to lyse cells and release virus. NP-40 was added to the infectious lysate to a final concentration of 0.1% before 3 rounds of centrifugation (3,300 × *g*, 10 min each, 4°C), each time discarding the pelleted cell debris. The clarified supernatant was loaded onto a 30% (w/v) sucrose cushion and subjected to ultracentrifugation at 150,000 × *g* (3 hours, 4°C). The resultant pellets were resuspended in PBS, then clarified by centrifugation at 17,000 × *g* (10 min) before being loaded onto a continuous 15%–60% sucrose gradient for ultracentrifugation at 300,000 × *g* (50 min, 4°C) and then fractionated. Peak fractions (determined by SDS-PAGE analysis) were combined and spun at 366,000 × *g* (60 min, 4°C), and the pellet was resuspended in PBS for a second round of sucrose gradient ultracentrifugation.

To remove sucrose for structural studies, peak fractions from the second sucrose gradient were combined and dialysed using a 10,000 molecular weight cutoff Slide-A-Lyzer dialysis cassette (Thermo Fisher Scientific, Waltham, MA, USA) in 1 L EM buffer (10 mM HEPES [pH 7.6], 200 mM NaCl, 5 mM MgCl_2_, 1 mM KCl, 1 mM CaCl_2_). After 1 hour at room temperature, the cassette was transferred into fresh EM buffer for another hour at room temperature before being transferred to fresh EM buffer for overnight incubation at 4°C. Virus was recovered from the dialysis cassette and stored at 4°C prior to imaging.

### TCID_50_ assay

To measure viral infectivity, TCID_50_ assays were performed according to a modified version of the protocol described by Hwang and colleagues [35]. RAW264.7 cells were seeded into 96-well plates at a density of 2.0 × 10^4^ cells/well and incubated for 24 hours. Subsequently, 10-fold serial dilutions of MNV in fresh DMEM were prepared, and 100 μl of each concentration was added to 100 μl of media already present in each well. For each concentration of MNV, 6 wells were infected. Cells were incubated for a further 72 hours before fixing with 4% paraformaldehyde (PFA) in PBS and staining with crystal violet solution to assess cytopathic effect. TCID_50_ values were calculated according to the Spearman and Kärber algorithm [36].

### PaSTRy assay

To investigate particle stability, PaSTRy assays [21] were performed according to the protocol described by Adeyemi and colleagues [37]. Briefly, 1.0 μg purified MNV was incubated in a 50 μl reaction mixture of 5 μM SYTO9, 150× SYPRO-Orange and PaSTRy buffer (2 mM HEPES [pH 8.0], 200 mM NaCl) on a temperature ramp from 25°C to 95°C. At every 1°C interval, the Stratagene MX3005p quantitative-PCR (qPCR) system was used to measure fluorescence.

### RNase protection assay

To investigate the disassembly of virus particles, an RNase protection assay was performed based on the protocol reported by Groppelli and colleagues [38], with several modifications. Briefly, purified MNV was heated to a range of temperatures. After heating, a fraction of each sample was retained for titration by TCID_50_ assay (‘Virus Infectivity’), whilst the remaining fraction was treated with RNase A (1 mg/ml) for 30 min at 37°C. To stop the reaction and extract viral RNA, TRIzol was added and RNA extracted using the Direct-zol RNA miniprep kit (Zymo Research, Irvine, CA, USA) according to the manufacturer’s instructions. Total extracted RNA was transfected into BHK-21 cells using lipofectin (as described previously [39]), supplemented with carrier RNA (yeast tRNA) to 1 μg per reaction. Forty-eight hours later, total virus was extracted by freeze–thaw, cell debris was clarified by centrifugation, and virus was titrated by TCID_50_ assay.

### Selection of hsMNV

To generate a thermally stable population of MNV, crude MNV samples were heated at 52°C for 30 min before cooling to 4°C. The surviving pool of virus was subsequently passaged at 37°C on RAW264.7 cells. Consecutive cycles of selection and passage were performed, after which the pool of virus was characterised.

### ELISAs

To check for changes to antigenicity, ELISAs were performed according to the protocol described by Hwang and colleagues [35]. Concentrated MNV suspended in PBS was used to coat ELISA wells overnight at 4°C, which were then washed with ELISA wash buffer (150 mM NaCl, 0.05% Tween 20) and blocked with ELISA blocking buffer (50 mM Na_2_CO_3_, 50 mM NaHCO_3_, 3% BSA [pH 11]) at 37°C for 2 hours. After blocking, wells were washed twice with ELISA wash buffer, then incubated with primary antibody (2D3 or 4F9) diluted 1:100 in ELISA III buffer (150 mM NaCl, 1 mM EDTA, 50 mM Tris-HCl, 0.05% Tween 20, 0.1% BSA [pH 11]) for 1 hour at 37°C. Primary antibody was removed, and wells were washed 4 times with ELISA wash buffer before adding secondary antibody (peroxidase-conjugated anti-mouse IgG [A9044, Sigma-Aldrich, St. Louis, MO, USA]) diluted 1:2,000 in ELISA III buffer for 1 hour at 37°C. Secondary antibody was removed, and wells were washed 4 times with ELISA wash buffer, then substrate (ABTS) was added and incubated at room temperature for 30–60 min. Reactions were stopped with 0.2 N phosphoric acid, and absorbance in each well was measured by plate reader at 415 nm.

### Cryo-electron microscopy

To prepare MNV samples for cryo-EM, lacey carbon 400-mesh copper grids coated with a <3-nm continuous carbon film (Agar Scientific, Stansted, UK) were glow-discharged in air or amylamine vapour (10 mA, 30 seconds) before applying two to three 3-μl aliquots of purified MNV to improve the concentration of virus on the grid surface (as described previously [40]). Each application was followed by a 30-second incubation period at 80% relative humidity (8°C), then the grid was manually blotted to remove excess fluid before the next application. Thirty seconds after the final application, grids were blotted and vitrified in liquid-nitrogen–cooled liquid ethane using a LEICA EM GP plunge freezing device (Leica Microsystems, Wetzlar, Germany). Grids were stored in liquid nitrogen prior to imaging with an FEI Titan Krios transmission electron microscope (ABSL, University of Leeds, Leeds, UK) at 300 kV, at a magnification of 75,000× and a calibrated object sampling of 1.065 Å/pixel. A complete set of data collection parameters for each sample is provided in S1 Table.

### Image processing

Following cryo-EM data collection, the RELION-2.1 and RELION-3.0 pipelines [41–43] were used for image processing. Drift correction was first performed on micrograph stacks using MOTIONCOR2 [44], and the contrast transfer function for each was estimated using Gctf [45]. A subset of virus particles was picked manually and subject to 2D classification, with the resultant classes used as templates for automatic particle picking [46]. Particles were classified through multiple rounds of reference-free 2D classification, and particles in poor quality classes were removed after each round. An initial 3D model was generated de novo [47] and used as a reference for 3D autorefinement with icosahedral symmetry imposed. This reconstruction was postprocessed to mask and correct for the B-factor of the map before (i) taking particles forward to CTF refinement and Bayesian polishing or (ii) further ‘clean-up’ by alignment-free 3D classification, with particles from subsequent 3D autorefinement and postprocessing being used for CTF refinement and Bayesian polishing. Multiple rounds of CTF refinement (with or without beamtilt refinement) and Bayesian polishing [48] were performed before final icosahedral-symmetry–imposed 3D autorefinement and postprocessing. The nominal resolution for each map was determined according to the ‘gold standard’ Fourier shell correlation (FSC) criterion (FSC = 0.143) [49], and the local resolution estimation tool in RELION was used to generate maps filtered by local resolution.

To investigate P domain mobility, a focussed 3D classification approach was employed (as described previously [16,50–52]). Briefly, each particle contributing to the final icosahedral-symmetry–imposed reconstruction was assigned 60 orientations corresponding to its icosahedrally related views using the relion_symmetry_expand tool. SPIDER [53] was used to generate a cylindrical mask, which was manually placed over the map to isolate either an AB-type or a CC-type P domain dimer, and the symmetry-expanded particles were subjected to masked 3D classification without alignment using a regularisation parameter (‘T’ number) of 20. Classes were inspected visually, and particles from selected classes (with assigned orientation information) were used to generate full capsid reconstructions without imposing symmetry using the relion_reconstruct tool.

### Model building and refinement

To generate a preliminary model for the VP1 asymmetric unit, the amino acid sequence corresponding to the S domain of MNV VP1 was used to build a homology model with the Phyre2 server [18], which was rigid-body fitted into each quasiequivalent position in the wtMNV density map using UCSF Chimera [54]. This preliminary model was manually refined in Coot [55], symmetrised in UCSF Chimera to generate the other 59 copies of the asymmetric unit that form the capsid, and then subject to ‘real space refinement’ in Phenix [56]. To improve the geometry of the coordinates and fit of the model to the density map, the S domain model was iterated between manual fitting in Coot and refinement in Phenix. Following this, the crystal structure of an MNV P domain complexed with CD300lf (PDB: 6C6Q) [13] was also fitted into the map to occupy each quasiequivalent position of the asymmetric unit after removing ligands/CD300lf and correcting the peptide sequence. P domain coordinates were combined with the refined S domain model and subjected to a single round of refinement in Phenix. For each real space refinement, secondary structure restraints were imposed. Molprobity [57] was used to validate the model.

### Analysis and visualisation

For structural analysis and generation of figures, density maps and atomic coordinates were viewed in UCSF Chimera [54], UCSF ChimeraX [58] and PyMOL (The PyMOL Molecular Graphics System, Version 2.0, Schrödinger, LLC). RMSD values between quasiequivalent states were calculated using the ‘MatchMaker’ tool of UCSF Chimera with default settings. Briefly, the fitted atomic coordinates for A-type, B-type, and C-type VP1 were aligned based on a subset of close-matching atom pairs (determined automatically by the ‘MatchMaker’ tool), and then the RMSD between A- and B-, A- and C-, or B- and C-type VP1 coordinates was calculated from variations in the CA atom position for each residue.

## Supporting information

S1 Fig Representative micrographs and FSC plots. Micrographs and FSC plots are given for wtMNV (A), hiMNV (B), and hsMNV (C) data sets. Scale bars show 100 nm. The resolution given for each data set is determined using the FSC = 0.143 criterion with high-resolution noise substitution to correct for overfitting (rlnFourierShellCorrelationCorrected) [59]. FSC, Fourier shell correlation; hiMNV, heat-inactivated MNV; hsMNV, heat-stable MNV; MNV, murine norovirus; wt, wild type.
(TIF)

S2 Fig Local resolution maps. Isosurface representations of (A) the 3.1-Å wtMNV reconstruction, (B) the 2.9-Å hiMNV reconstruction, and (C) 3.1-Å hsMNV reconstruction are shown, coloured according to local resolution. All reconstructions are shown at 1 σ. hiMNV, heat-inactivated MNV; hsMNV, heat-stable MNV; MNV, murine norovirus; wt, wild type.
(TIF)

S3 Fig Representative EM density. Representative EM densities are given from S domain and P1 and P2 (sub)domains for each MNV reconstruction. The coordinates for a C-type monomer of wtMNV VP1 rigid-body fitted into each map are shown in each case. Local resolution-coloured density is also shown. EM, electron microscopy; MNV, murine norovirus; P domain, protruding domain; S domain, shell domain; wt, wild type.
(TIF)

S4 Fig Focussed classification of wtMNV. (A,B) All classes resulting from focussed classification of (A) AB P domain dimers and (B) CC P domain dimers from wtMNV, shown at 2 σ and coloured according to height. Classes with an inverted *Z* orientation are indicated by a dashed blue box. The proportion of P dimers in each class is given. (C) Reconstructed capsid from a single AB P domain dimer class (shown by the red box in (A)) shown at 2.8 σ with a radial colour scheme. The area used for focussed classification is indicated by a red circle. The 5-fold (hexagon), 3-fold (triangle), and 2-fold (oval) icosahedral axes are indicated. MNV, murine norovirus; P domain, protruding domain; wt, wild type.
(TIF)

S5 Fig Comparison of unliganded wtMNV VP1 and bile-salt–conjugated wtMNV VP1 (PDB: 6P4J, EMDB: 20250) from Sherman and colleagues [10]. In each case, both density maps and the atomic coordinates for all quasiconformers of wtMNV VP1, coloured according to relative B-factor (6S6L: 0 to 20 Å^2^, 6P4J: 0 to 150 Å^2^), are shown. Overlays of density maps and atomic coordinates are also shown (grey: wtMNV, purple: wtMNV + GCDCA). EMDB, Electron Microscopy Data Bank; GCDCA, glychochenodeoxycholic acid; MNV, murine norovirus; PDB, Protein Data Bank; VP, viral protein; wt, wild type.
(TIF)

S6 Fig Focussed classification of hiMNV CC-type P domain dimers. All focussed classes are shown at 2 σ with the proportion of CC-type P dimers assigned to each class. hiMNV, heat-inactivated MNV; MNV, murine norovirus; P domain, protruding domain.
(TIF)

S7 Fig Related to hsMNV. (A) wtMNV (grey) and hsMNV (orange) were incubated for 30 min at a range of different temperatures, then titred by TCID_50_ assay on RAW264.7 cells (*n* = 3 ± SEM). (B) Stereo views of atomic coordinates for VP1 fitted into wtMNV (grey) or hsMNV (orange), with S domains shown in green. The AB-type P domain dimer and C-type P domain were fitted separately, then refined together. The mutated residue is shown as magenta (wtMNV, L412) or dark purple (hsMNV, L412Q) spheres. (C) All focussed classes from focussed classification of hsMNV CC-type P domain dimers, shown at 2 σ. The proportion of all P dimers assigned to each class is given. The class marked by the blue dashed box was inverted in the Z plane. hsMNV, heat-stable MNV; MNV, murine norovirus; P domain, protruding domain; S domain, shell domain; TCID_50_, median tissue culture infectious dose; VP, viral protein; wt, wild type.
(TIF)

S8 Fig Norovirus VP1 sequence alignment. Alignment of VP1 sequences from norovirus genogroups GV (NCBI reference sequence: YP_720002.1), GI (NP_056821.2), GII (YP_009237898.1), GIII (YP_009237901.1), and GIV (YP_009237904.1). The MNV (GV) VP1 sequence is given in the top position. Sequences were aligned using Clustal Omega with default parameters [60,61,62] and are shown with the Clustal colouring scheme. MNV (GV) VP1 L412 is indicated by the red asterisk. Approximate positions for the P1 and P2 subdomains are indicated by red and purple dashed lines, respectively. MNV, murine norovirus; P domain, protruding domain; VP, viral protein.
(TIF)

S1 Table Data collection and image processing parameters. (DOCX)

S2 Table Model building and validation. (DOCX)

S1 Movie Differences in P domain conformation between wtMNV from Sherman and colleagues [10] and this work. Atomic coordinates for the MNV VP1 P domain were rigid-body fitted into the apo wtMNV EM density map from Sherman and colleagues (EMDB-20252 [10]) or the apo wtMNV map reported here after low-pass filtering both to 4.0-Å resolution. AB-type P dimers and C-type P domains were fitted separately. The UCSF Chimera tool ‘Morph Conformations’ was used to switch between these 2 conformations. P domains are shown in orange. S domains (green) are also shown for clarity. EMDB, Electron Microscopy Data Bank; MNV, murine norovirus; P domain, protruding domain; S domain, shell domain; wt, wild type.
(MOV)

S2 Movie hsMNV has ‘twisted’ AB-type P domains. Morphing from the wtMNV (grey) to the hsMNV (orange) density maps highlights the change in AB-type P domain orientation. Maps are low-pass filtered to 8.0 Å for clarity and shown at 1.5 σ with the back plane clipped to remove the S domains. hsMNV, heat-stable MNV; MNV, murine norovirus; P domain, protruding domain; S domain, shell domain; wt, wild type.
(MOV)

S3 Movie An interface is disrupted between P domain dimers in hsMNV. The atomic coordinates for wtMNV VP1 were separated into components (S domains, C-type P domain, and AB-type P domain dimer) and rigid-body fitted, then refined into the hsMNV density map. The change between these 2 conformations is shown, with P domains shown in orange, S domains in green, and the mutated residue (L412Q) highlighted in cyan. Density maps (wtMNV: grey, hsMNV: orange) are shown at 2 σ after low-pass filtering to 5.0 Å resolution. hsMNV, heat-stable MNV; MNV, murine norovirus; P domain, protruding domain; S domain, shell domain; wt, wild type.
(MOV)

S1 Data Numerical data for Fig 2. Sheet 1 shows the TCID_50_ titres for wtMNV used to plot Fig 2A. Sheet 2 shows the SYTO-9 and SYPRO-Orange fluorescence for wtMNV used to plot Fig 2B. The data were normalised on a scale between the fluorescence value at 25°C (set to 0%) and the highest fluorescence value (set to 100%) for each repeat. At least 2 technical repeats were performed for each assay, and the means of these normalised values were taken for each biological repeat. The means of the biological repeats were plotted in Fig 2B. Sheet 3 shows the TCID_50_ titres for virus or RNA used to plot Fig 2C. The log_10_ value of each titre was calculated, then log_10_ values were normalised against the titre at 37°C. The mean normalised titres at each temperature were plotted in Fig 2C. MNV, murine norovirus; TCID_50_, median tissue culture infectious dose; wt, wild type.
(XLSX)

S2 Data Numerical data for Fig 4. Sheet 1 shows the TCID_50_ titres used to plot Fig 4A. Sheet 2 shows the TCID_50_ titres of wtMNV and MNV52 used to plot Fig 4B. The log_10_ value of each titre was calculated, then log_10_ values were normalised against the average titre at 37°C for each condition. The mean normalised titres at each temperature were plotted in Fig 4B. Sheet 3 shows the SYTO-9 absorbance values for wtMNV and for MNV52 used to plot Fig 4C. Each value was normalised against the smallest and largest mean values for each condition, and the means of normalised values were used to generate the trace in Fig 4C. Sheet 4 shows absorbance values for the 2 monoclonal antibodies, 2D3 and 4F9, for both wtMNV and hsMNV, used to plot Fig 4D. Values were normalised by subtracting the average absorbance value of a BSA control, and the mean normalised values were plotted in Fig 4D. hsMNV, heat-stable MNV; MNV, murine norovirus; TCID_50_, median tissue culture infectious dose; wt, wild type.
(XLSX)

S3 Data Numerical data for panel A in S7 Fig. TCID_50_ titres comparing wtMNV and hsMNV after 30-min incubation at the given temperature. Normalised values were calculated from these data by dividing each value by the average titre at 37°C (wtMNV = 1.54 × 10^5^, hsMNV = 3.05 × 10^6^). The mean normalised values were plotted in S7A Fig. hsMNV, heat-stable MNV; MNV, murine norovirus; TCID_50_, median tissue culture infectious dose; wt, wild type.
(XLSX)

## Abbreviations

BHK: baby hamster kidney
CD300lf: cluster of differentiation 300-like family member f
cryo-EM: cryo-electron microscopy
DMEM: Dulbecco’s Modified Eagle’s Medium
FMDV: foot-and-mouth disease virus
FSC: Fourier shell correlation
GCDCA: glychochenodeoxycholic acid
hiMNV: heat-inactivated MNV
hsMNV: heat-stable MNV
MNV: murine norovirus
MOI: multiplicity of infection
P domain: protruding domain
PaSTRy: Particle Stability Thermal Release
PBS: phosphate-buffered saline
PDB: Protein Data Bank
PDBePISA: Protein Data Bank in Europe Protein Interfaces, Surfaces and Assemblies
PFA: paraformaldehyde
qPCR: quantitative PCR
RMSD: root mean-square deviation
RT-PCR: reverse transcriptase-PCR
S domain: shell domain
TCA: taurocholic acid
TCID_50_: median tissue culture infectious dose
VLP: virus-like particle
VPg: viral protein genome-linked
wt: wild type
