# Supplementary figures and images for: Dynamics in the murine norovirus capsid revealed by high-resolution cryo-EM
Source: PLoS Biol. 2020 Mar 31;18(3):e3000649. doi: 10.1371/journal.pbio.3000649 (PMC7108717; doi:10.1371/journal.pbio.3000649)

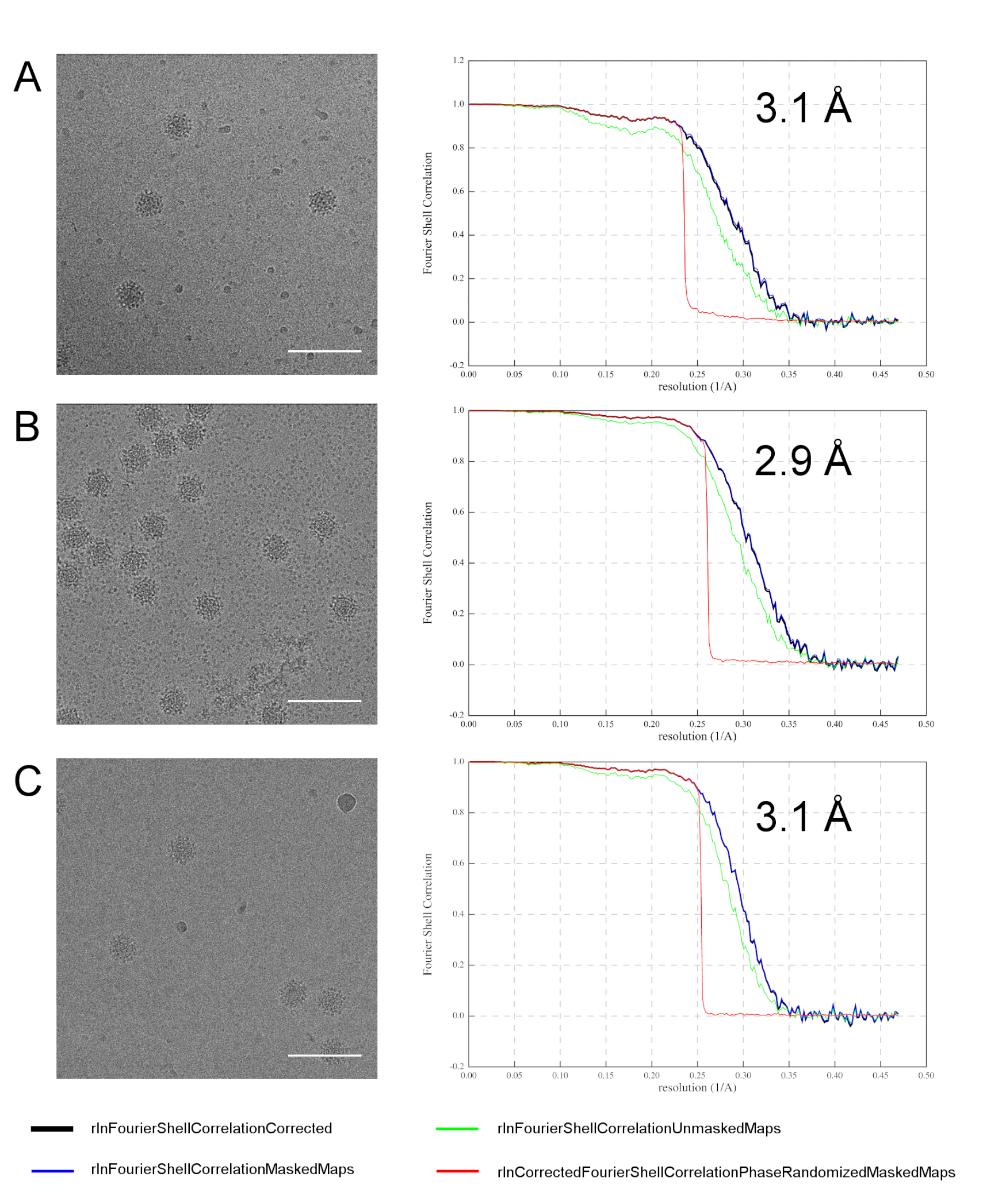

Supplement: S1 Fig — Micrographs and FSC plots are given for wtMNV (A), hiMNV (B), and hsMNV (C) data sets. Scale bars show 100 nm. The resolution given for each data set is determined using the FSC = 0.143 criterion with high-resolution noise substitution to correct for overfitting (rlnFourierShellCorrelationCorrected) [59]. FSC, Fourier shell correlation; hiMNV, heat-inactivated MNV; hsMNV, heat-stable MNV; MNV, murine norovirus; wt, wild type. (TIF) [file pbio.3000649.s001.tif]

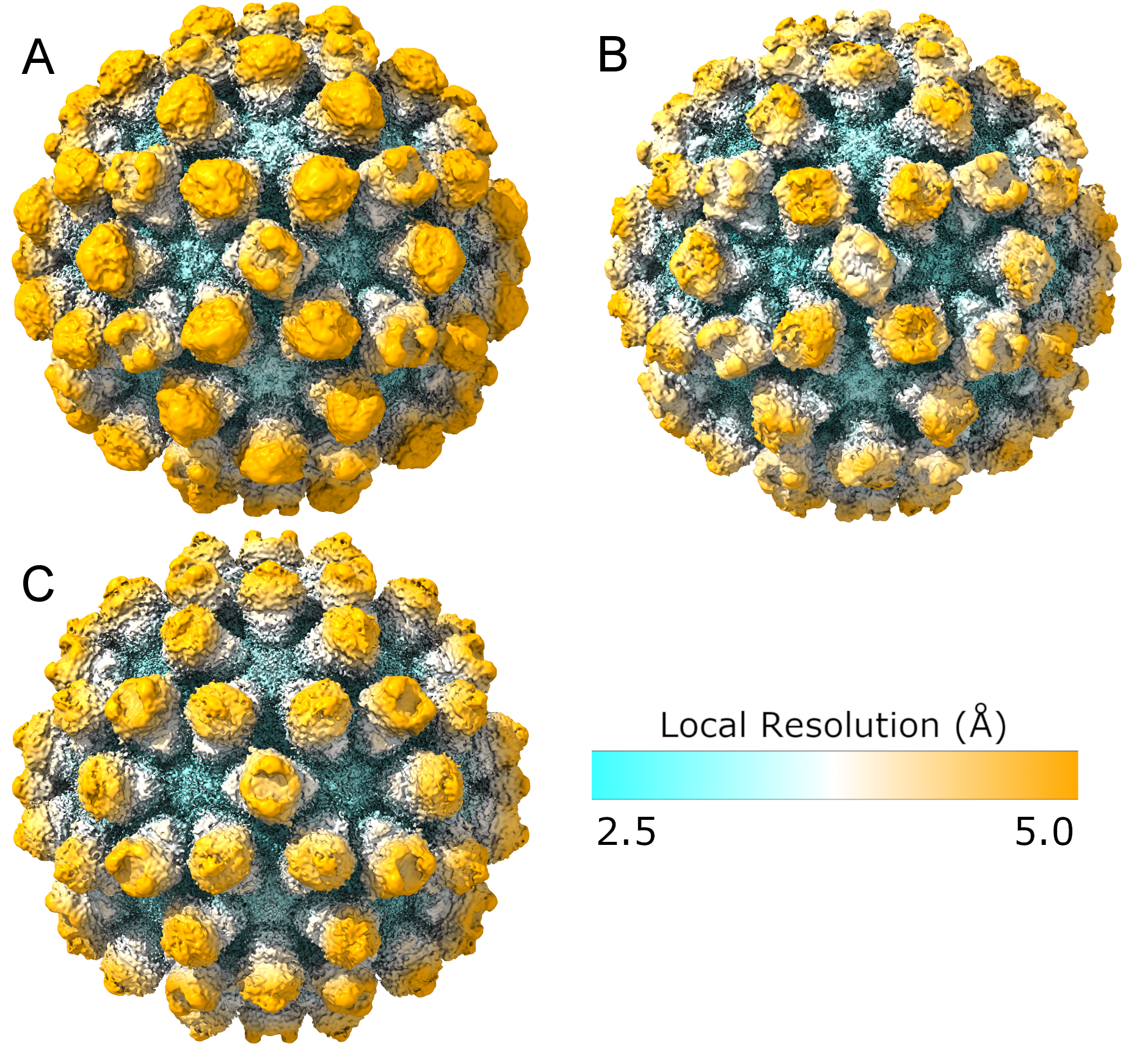

Supplement: S2 Fig — Isosurface representations of (A) the 3.1-Å wtMNV reconstruction, (B) the 2.9-Å hiMNV reconstruction, and (C) 3.1-Å hsMNV reconstruction are shown, coloured according to local resolution. All reconstructions are shown at 1 σ. hiMNV, heat-inactivated MNV; hsMNV, heat-stable MNV; MNV, murine norovirus; wt, wild type. (TIF) [file pbio.3000649.s002.tif]

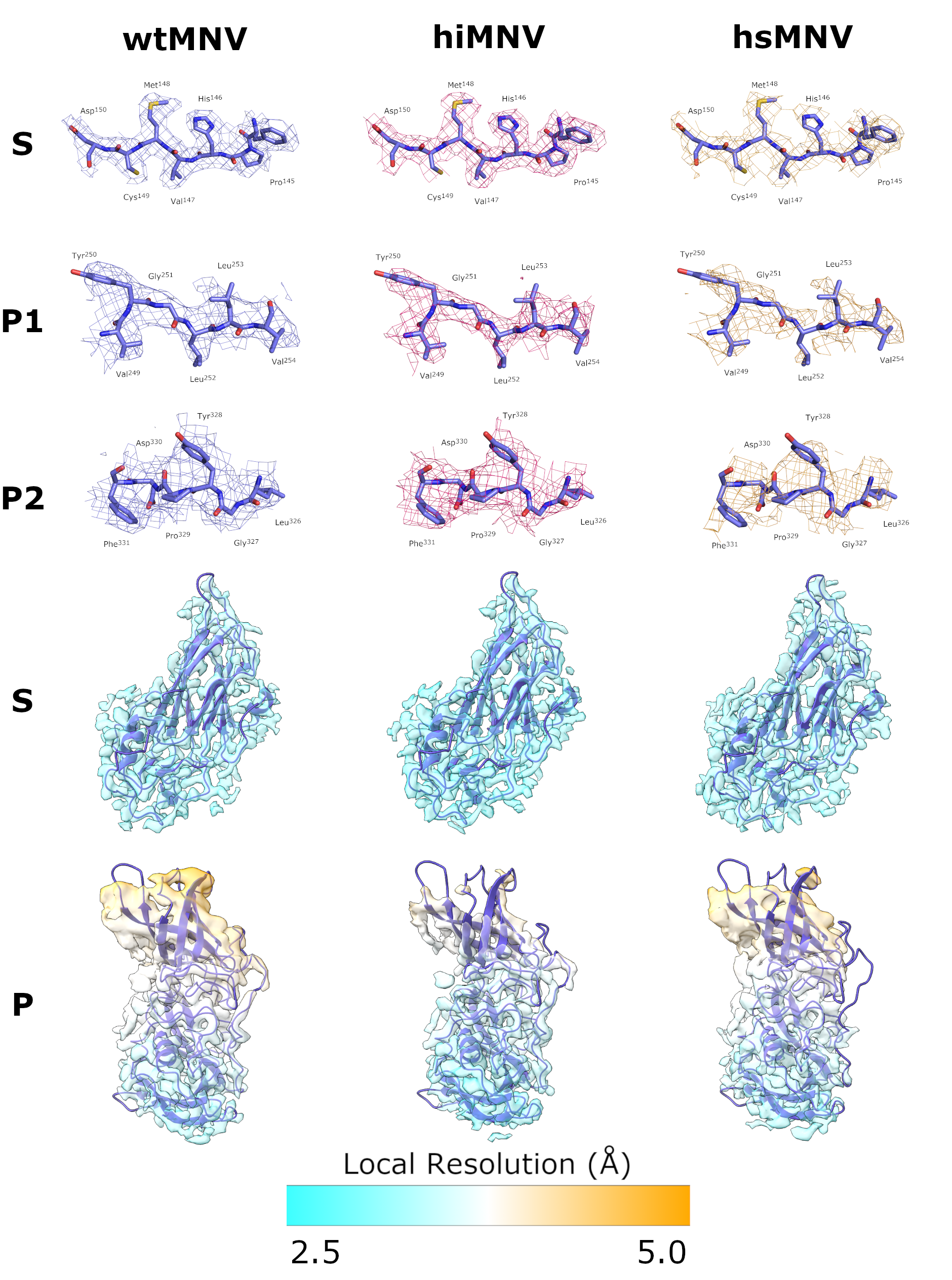

Supplement: S3 Fig — Representative EM densities are given from S domain and P1 and P2 (sub)domains for each MNV reconstruction. The coordinates for a C-type monomer of wtMNV VP1 rigid-body fitted into each map are shown in each case. Local resolution-coloured density is also shown. EM, electron microscopy; MNV, murine norovirus; P domain, protruding domain; S domain, shell domain; wt, wild type. (TIF) [file pbio.3000649.s003.tif]

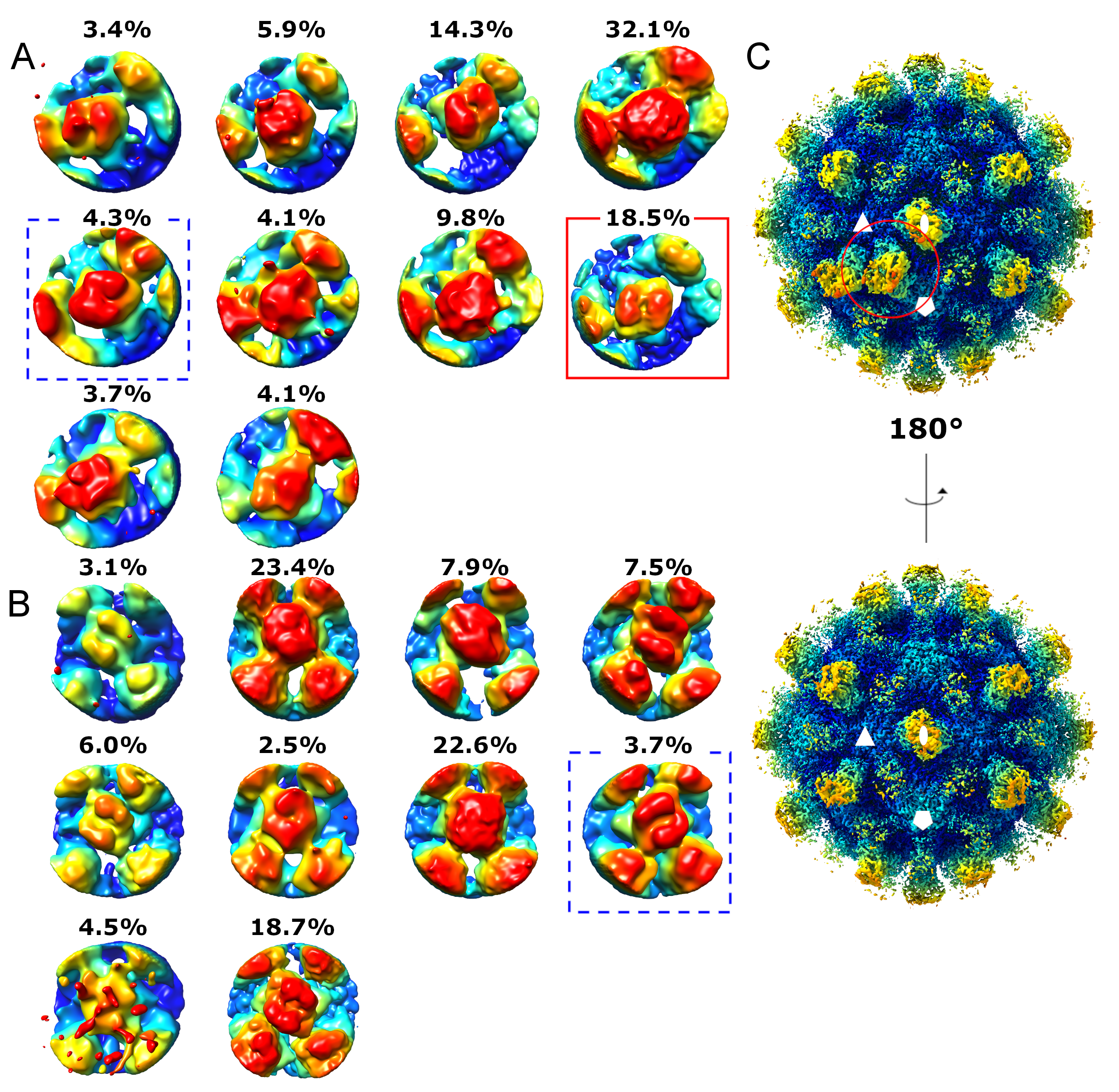

Supplement: S4 Fig — (A,B) All classes resulting from focussed classification of (A) AB P domain dimers and (B) CC P domain dimers from wtMNV, shown at 2 σ and coloured according to height. Classes with an inverted Z orientation are indicated by a dashed blue box. The proportion of P dimers in each class is given. (C) Reconstructed capsid from a single AB P domain dimer class (shown by the red box in (A)) shown at 2.8 σ with a radial colour scheme. The area used for focussed classification is indicated by a red circle. The 5-fold (hexagon), 3-fold (triangle), and 2-fold (oval) icosahedral axes are indicated. MNV, murine norovirus; P domain, protruding domain; wt, wild type. (TIF) [file pbio.3000649.s004.tif]

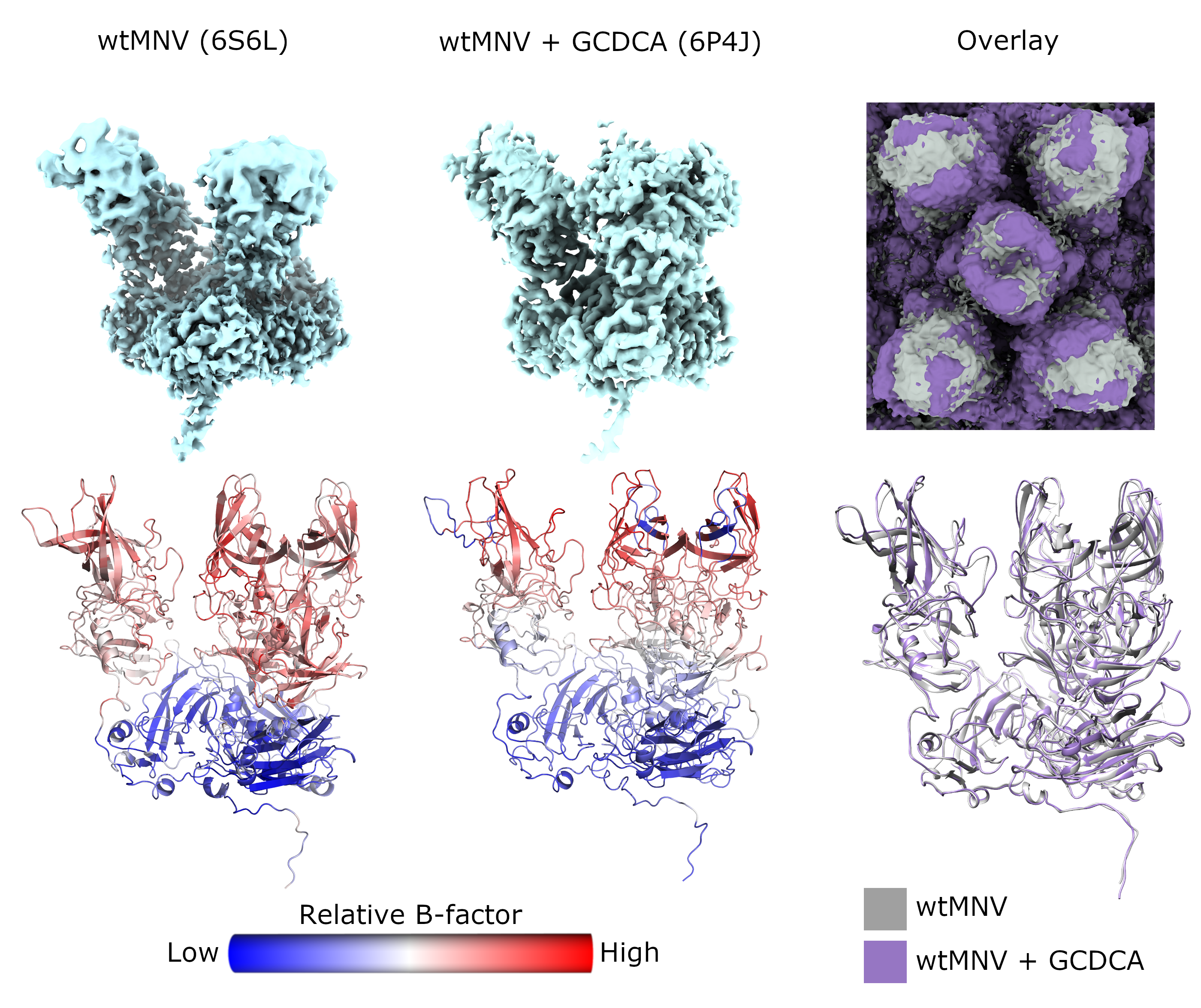

Supplement: S5 Fig — In each case, both density maps and the atomic coordinates for all quasiconformers of wtMNV VP1, coloured according to relative B-factor (6S6L: 0 to 20 Å2, 6P4J: 0 to 150 Å2), are shown. Overlays of density maps and atomic coordinates are also shown (grey: wtMNV, purple: wtMNV + GCDCA). EMDB, Electron Microscopy Data Bank; GCDCA, glychochenodeoxycholic acid; MNV, murine norovirus; PDB, Protein Data Bank; VP, viral protein; wt, wild type. (TIF) [file pbio.3000649.s005.tif]

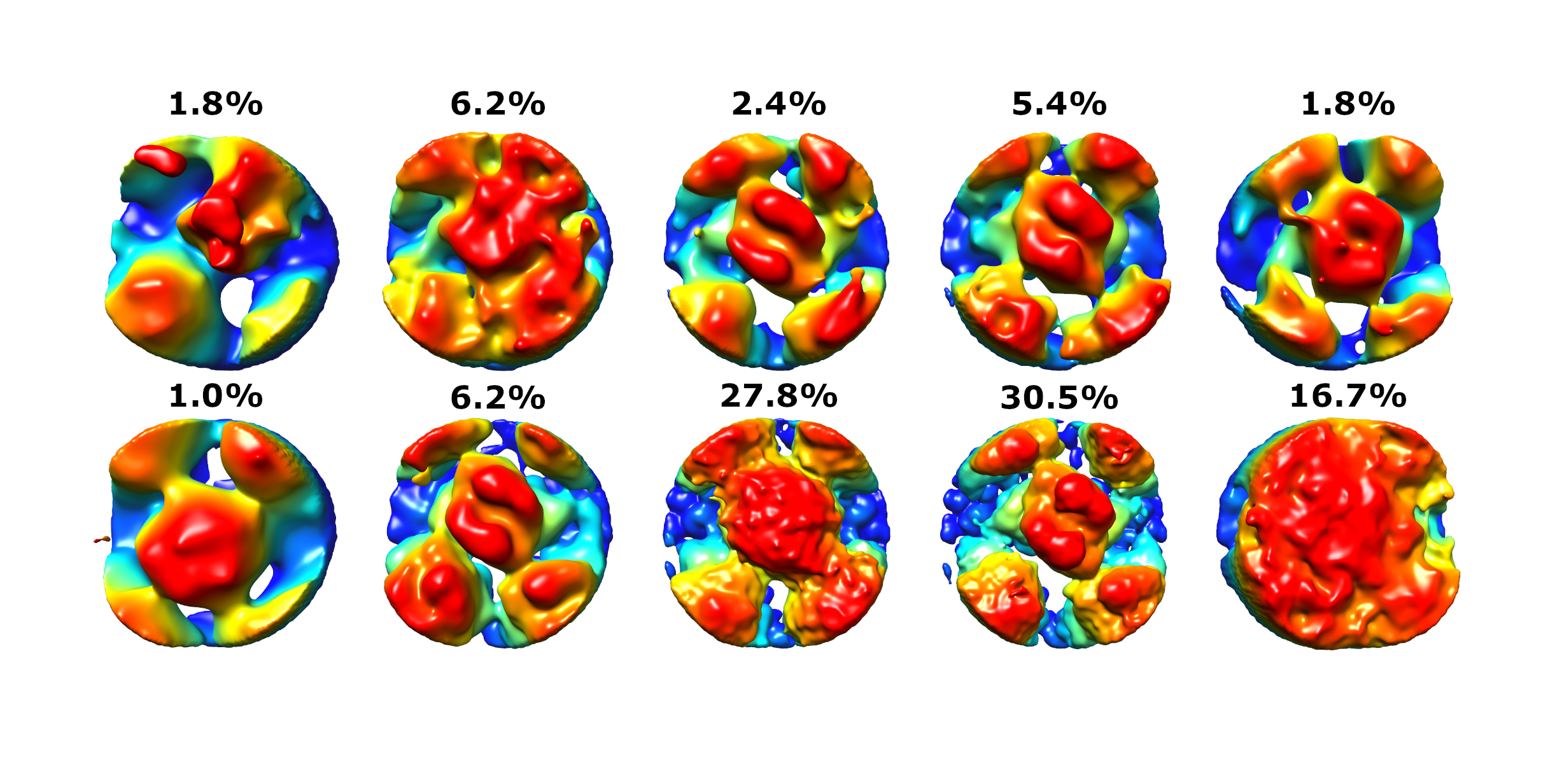

Supplement: S6 Fig — All focussed classes are shown at 2 σ with the proportion of CC-type P dimers assigned to each class. hiMNV, heat-inactivated MNV; MNV, murine norovirus; P domain, protruding domain. (TIF) [file pbio.3000649.s006.tif]

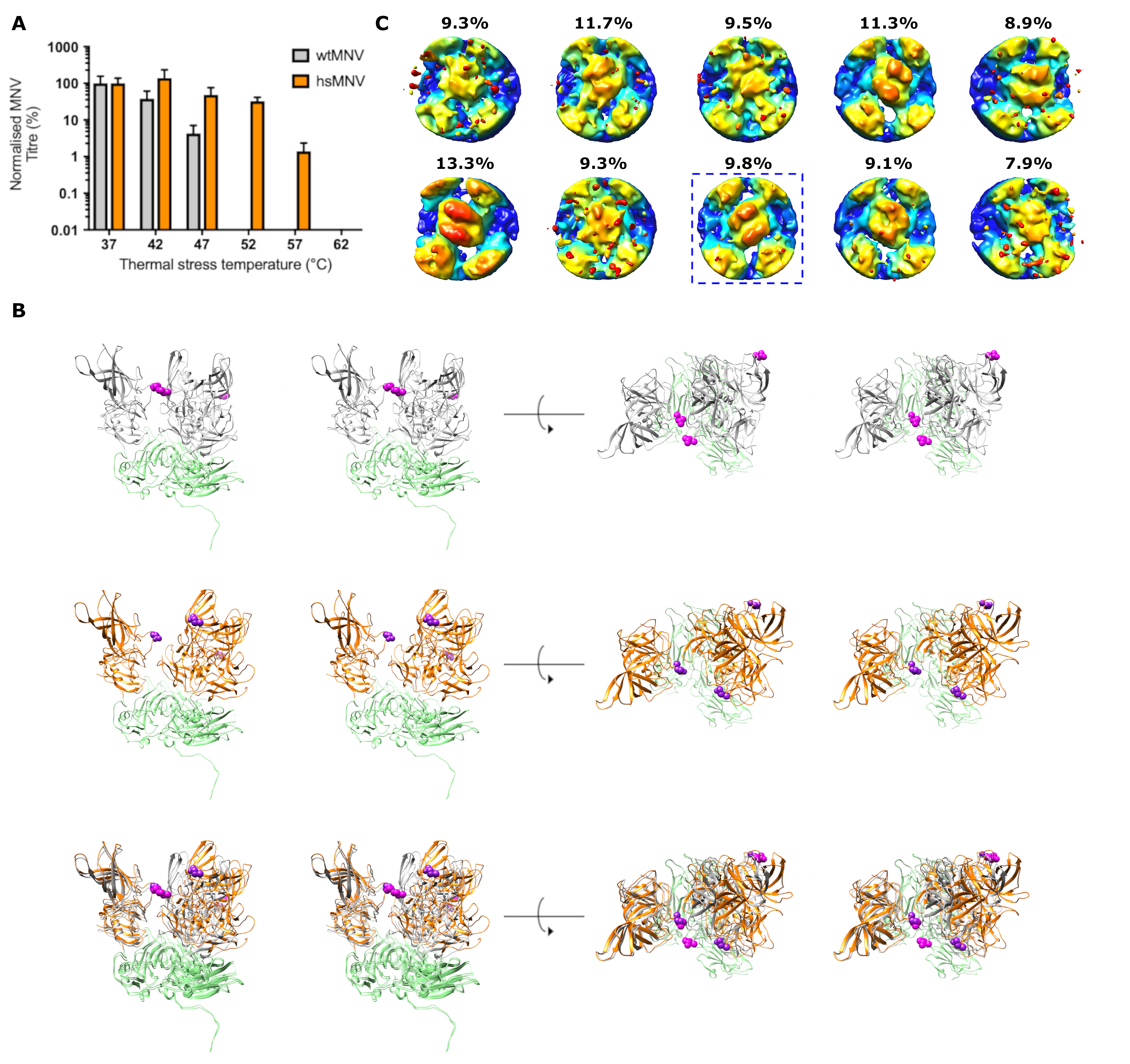

Supplement: S7 Fig — (A) wtMNV (grey) and hsMNV (orange) were incubated for 30 min at a range of different temperatures, then titred by TCID50 assay on RAW264.7 cells (n = 3 ± SEM). (B) Stereo views of atomic coordinates for VP1 fitted into wtMNV (grey) or hsMNV (orange), with S domains shown in green. The AB-type P domain dimer and C-type P domain were fitted separately, then refined together. The mutated residue is shown as magenta (wtMNV, L412) or dark purple (hsMNV, L412Q) spheres. (C) All focussed classes from focussed classification of hsMNV CC-type P domain dimers, shown at 2 σ. The proportion of all P dimers assigned to each class is given. The class marked by the blue dashed box was inverted in the Z plane. hsMNV, heat-stable MNV; MNV, murine norovirus; P domain, protruding domain; S domain, shell domain; TCID50, median tissue culture infectious dose; VP, viral protein; wt, wild type. (TIF) [file pbio.3000649.s007.tif]

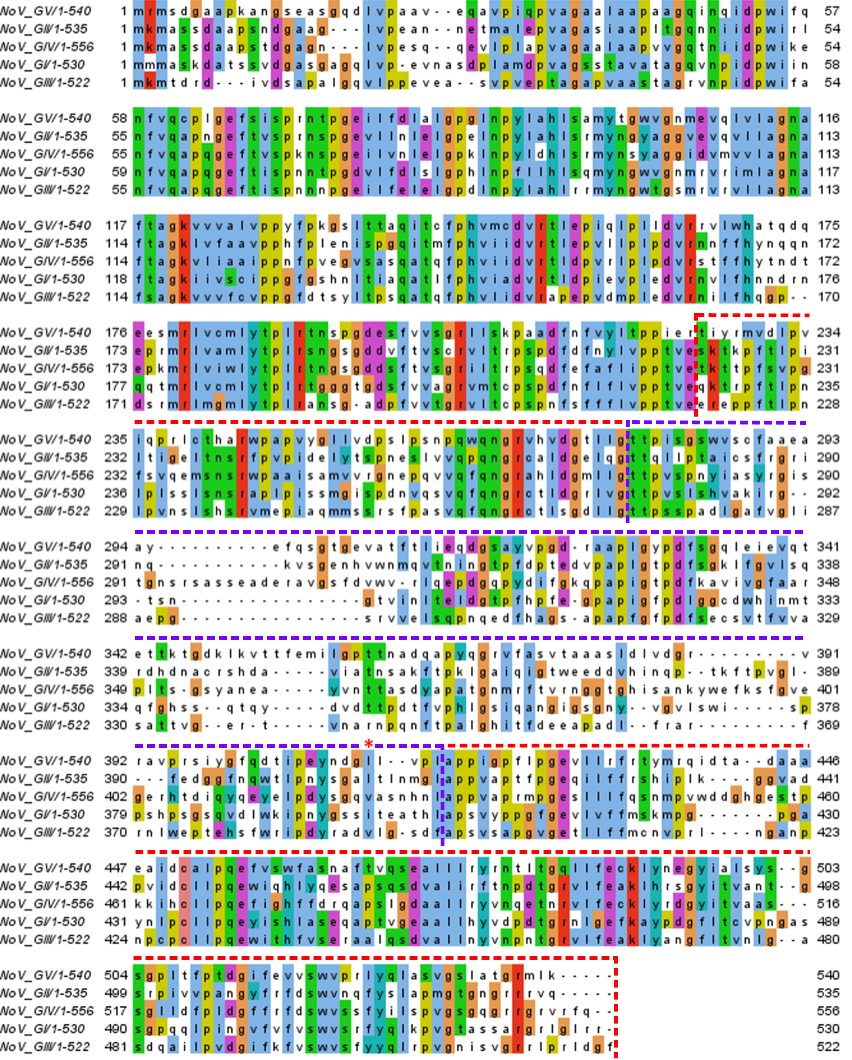

Supplement: S8 Fig — Alignment of VP1 sequences from norovirus genogroups GV (NCBI reference sequence: YP_720002.1), GI (NP_056821.2), GII (YP_009237898.1), GIII (YP_009237901.1), and GIV (YP_009237904.1). The MNV (GV) VP1 sequence is given in the top position. Sequences were aligned using Clustal Omega with default parameters [60,61,62] and are shown with the Clustal colouring scheme. MNV (GV) VP1 L412 is indicated by the red asterisk. Approximate positions for the P1 and P2 subdomains are indicated by red and purple dashed lines, respectively. MNV, murine norovirus; P domain, protruding domain; VP, viral protein. (TIF) [file pbio.3000649.s008.tif]
