# Supplementary material for: Dynamics in the murine norovirus capsid revealed by high-resolution cryo-EM
Source: PLoS Biol. 2020 Mar 31;18(3):e3000649. doi: 10.1371/journal.pbio.3000649 (PMC7108717; doi:10.1371/journal.pbio.3000649)
Supplement: S1 Table — (DOCX) [file pbio.3000649.s009.docx]

|  | **wtMNV** | **hiMNV** | **hsMNV** |
| --- | --- | --- | --- |
| Microscope | FEI Titan Krios | FEI Titan Krios | FEI Titan Krios |
| Camera | Falcon III | Falcon III | Falcon III |
| Voltage (kV) | 300 | 300 | 300 |
| Pixel size (Å) | 1.065 | 1.065 | 1.065 |
| Total dose (e^-^/Å^2^) | 59 | 59 | 64 |
| Number of frames | 59 | 59 | 59 |
| Defocus range (µm) | -0.5 to -2.9 | -0.5 to -2.9 | -0.7 to -3.0 |
| Number of micrographs | 13692 | 2620 | 7617 |
| Acquisition software | FEI EPU | FEI EPU | FEI EPU |
| Motion correction | MotionCor2 | MotionCor2 | MotionCor2 |
| CTF estimation | GCTF | GCTF | GCTF |
| Image processing | Relion 3.0 | Relion 3.0 | Relion 3.0 |
| Particles contributed | 7811 | 14266 | 35263 |
| B-factor | -129 | -136 | -153 |
| Resolution (FSC=0.143) | 3.1 | 2.9 | 3.1 |
