# Supplementary material for: Dynamics in the murine norovirus capsid revealed by high-resolution cryo-EM
Source: PLoS Biol. 2020 Mar 31;18(3):e3000649. doi: 10.1371/journal.pbio.3000649 (PMC7108717; doi:10.1371/journal.pbio.3000649)
Supplement: S2 Table — (DOCX) [file pbio.3000649.s010.docx]

| Model | **wtMNV VP1** |
| --- | --- |
| PDB ID | 6S6L |
| Residues modelled  (from S domain homology model) | **A**: 20‑191; 199‑222 **B**: 17‑221 **C**: 30‑227 |
| Residues modelled  (from PDB: 6C6Q) | **A**: 228-531 **B**: 229-531 **C**: 228-531 |
| RMSD |  |
| *Bond lengths (Å)* | 0.0139 |
| *Bond angles (° )* | 1.42 |
| Validation |  |
| *All-atom clashscore* | 5.43 |
| *MolProbity score* | 1.66 |
| *Rotamer outliers (%)* | 0.16 |
| Ramachandran plot |  |
| *Favoured (%)* | 94.73 |
| *Allowed (%)* | 5.27 |
| *Outliers (%)* | 0.00 |
